# The dual roles of ferroptosis in digestive tract tumors: mechanisms, microenvironment regulation, and therapeutic integration with emphasis on immune interactions

**DOI:** 10.3389/fimmu.2026.1737847

**Published:** 2026-01-30

**Authors:** Jiaojiao Guo, Ziqing Wang, Yuhan Zhang, Chenyu Xie, Yulong Chen, Liangliang Ma, Zhizhong Guo, Congcong Zhang

**Affiliations:** 1 Henan University of Chinese Medicine, Zhengzhou, Henan, China; 2 The Second Clinical Medical College of Henan University of Chinese Medicine, Zhengzhou, Henan, China; 3 School of Rehabilitation Sciences Henan University of Chinese Medicine, Zhengzhou, Henan, China; 4 Rehabilitation Center, The First Affiliated Hospital of Henan University of Chinese Medicine, Zhengzhou, Henan, China

**Keywords:** digestive tract cancer, ferroptosis, GPX4, immunotherapy, radiotherapy sensitization, SLC7A11, therapeutic

## Abstract

Malignant tumors of the digestive tract are a major global health burden, characterized by high incidence and mortality rates, limited treatment options for advanced patients, and poor prognosis. Ferroptosis is an iron-regulated form of cell death driven by lipid peroxide (LPO) accumulation, and it is closely linked to the occurrence and progression of various cancers. Ferroptosis plays a critical role in the proliferation, metastasis, drug resistance, and microenvironment regulation of digestive tract cancer. This article will systematically examine the dual roles of ferroptosis through the core concepts of mechanism analysis, microenvironment regulation, and immune interactions, while exploring the therapeutic potential of targeting ferroptosis in the treatment of gastrointestinal malignancies.

## 1 Introduction

Digestive tract tumors refer to malignant tumors that occur in the digestive tract and its accessory organs. According to the location of occurrence, they can be classified into the upper digestive tract, lower digestive tract, liver, gallbladder, and pancreatic system, among others. According to GLOBOCAN 2022 data, approximately 4.906 million new cases of digestive tract cancer were reported globally in 2022, accounting for 24.6% of all new cancer cases. The number of deaths caused by digestive tract cancers has reached 3.602 million, accounting for 33.2% of the total global cancer deaths, significantly higher than the mortality rates of other organ systems, including stomach cancer, liver cancer, and colorectal cancer (CRC) (1, 2). Although the wide application of endoscopic screening has improved the early diagnosis rate of certain cancer types, the insidious early symptoms and rapid progression of digestive tract tumors, as well as the limited treatment options in the advanced stage, jointly lead to a five-year survival rate of less than 30% (3). Currently, the standard treatment primarily consists of surgical intervention, supplemented by radiotherapy, chemotherapy, targeted therapy, and immunotherapy. The overall objective response rate remains below 20% (4), and facing major challenges including immune-related toxicity, heterogeneity of the tumor immune microenvironment, and acquired drug resistance. Therefore, exploring new mechanisms of cell death to expand treatment options has become the core direction of digestive tract cancer research.

Ferroptosis was first discovered by Dixon et al. in 2012 (5). Unlike the classical mode of cell death, it is characterized by the accumulation of LPO dependent on iron (5). Existing studies have shown that ferroptosis is associated with a variety of diseases, including neurodegenerative diseases, cardiovascular diseases, and various types of cancer (6). Because ferroptosis can selectively eliminate cancer cells, the induction of ferroptosis has attracted considerable attention from scientists in the field of oncology. This method can not only exert therapeutic effects independently but also be used in combination with existing targeted drugs or immune checkpoint inhibitors (ICB), thereby significantly enhancing the overall therapeutic effect (7).

However, the immune response of tumor cells to ferroptosis remains poorly understood, and the views of the academic community are quite diverse and complex (7, 8). This review aims to systematically elucidate the molecular mechanisms of ferroptosis. It focuses on the biological functions and regulatory networks of ferroptosis in digestive tract malignancies. The review adopts a three-pronged approach: mechanism-based analysis forms the foundation, microenvironmental regulation is the core, and immune interactions serve as an extension. In addition, this article explores the synergistic treatment potential of combining ferroptosis with ICB and systemic drugs. It also examines multiple strategies for targeting ferroptosis to enhance tumor sensitivity to traditional radiotherapy and chemotherapy. The ultimate goal is to provide forward-looking insights and a scientific basis for the clinical transformation and application prospects of ferroptosis as an innovative treatment strategy for digestive tract cancers.

## 2 Molecular mechanisms of ferroptosis

**Figure 1:**
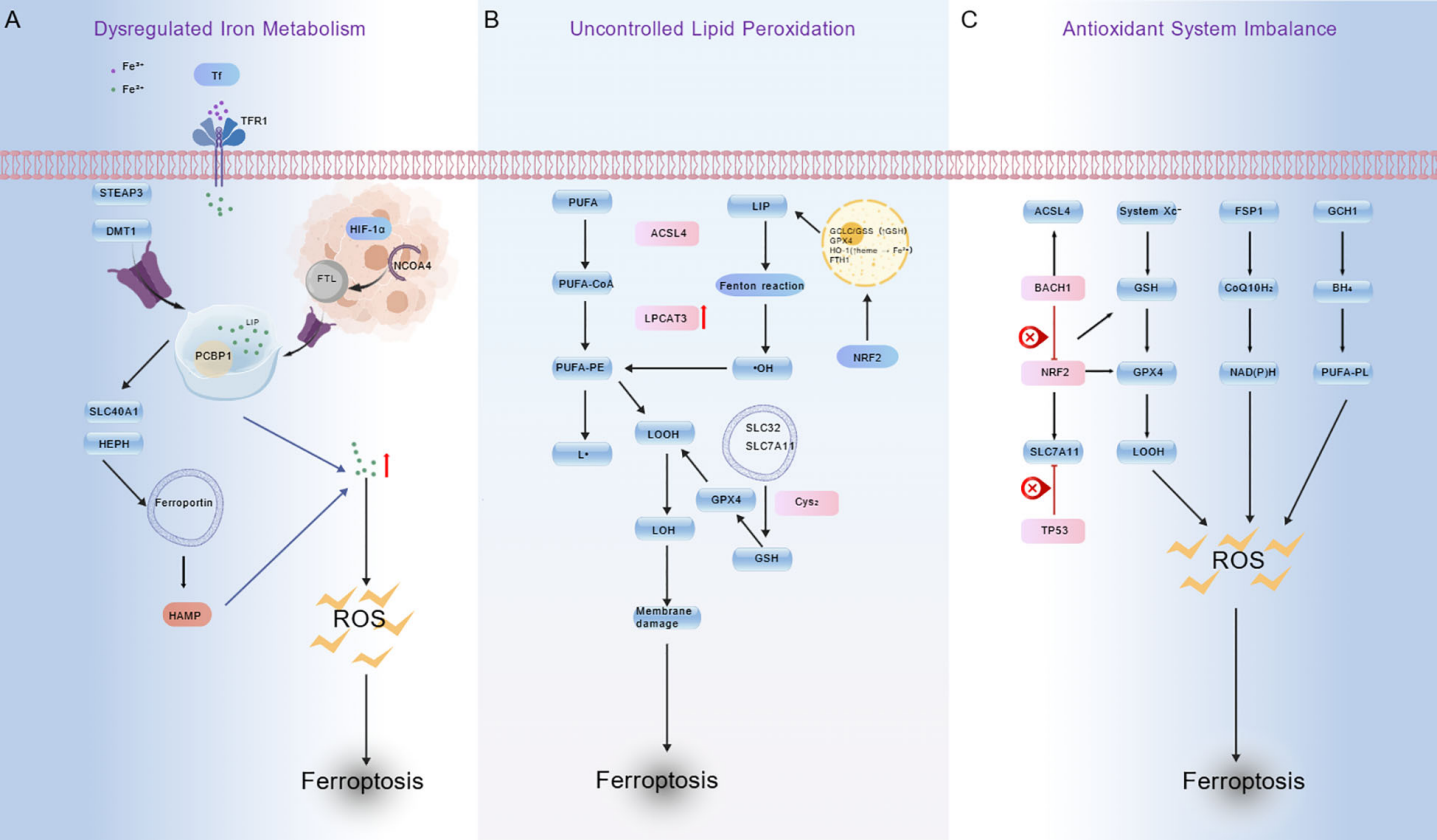
Schematic diagram of the three major pathways involved in ferroptosis. **(A)** represents the iron metabolism dysfunction pathway, showing how abnormalities in iron metabolism-related molecules lead to reactive oxygen species (ROS) accumulation, thereby triggering ferroptosis; **(B)** The uncontrolled lipid peroxidation pathway, depicting how lipid peroxidation involving polyunsaturated fatty acid (PUFA) metabolism-related molecules drives ferroptosis; **(C)** The antioxidant system imbalance pathway, illustrating how abnormalities in antioxidant-related molecules cause ROS accumulation and trigger ferroptosis. Created with (9).

Ferroptosis is a unique form of cell death, distinct from the classic forms of apoptosis, necrosis, or pyroptosis. Ferroptosis mainly depends on the regulation of iron elements. The core molecular mechanism primarily involves three key links: the first is the disruption of iron metabolism, the second is uncontrolled lipid peroxidation, and the third is an imbalance in the cellular antioxidant defense system. These three links are interrelated and together cause ferroptosis (**Figure 1**).

### 2.1 Dysregulated iron metabolism

Disorders of iron metabolism are key factors in initiating ferroptosis. Elevated intracellular free iron levels promote the Fenton reaction, accelerating lipid peroxidation. When extracellular Fe³^+^ binds to transferrin (Tf), it enters the cell through endocytosis mediated by transferrin receptor 1 (TfR1). This mechanism enhances iron uptake. *In vivo*, the metal reductase Six-Transmembrane Epithelial Antigen of Prostate 3 (STEAP3) converts Fe^3+^ to Fe^2+^. The Fe^2+^ is then transported to the labile iron pool (LIP) in the cytoplasm via divalent metal transporter 1 (DMT1). As a result, intracellular iron levels increase (10, 11).

Cancer cells generally have abnormal iron storage capacity, which leads to an enhanced release mechanism. Hypoxia-inducible factor 1α (HIF-1α) promotes the expression of ferritin light chain (FTL), thereby enhancing iron storage and boosting resistance to ferroptosis. Nuclear Receptor Coactivator 4 (NCOA4) promotes ferritin autophagy, degrades ferritin, and releases a large amount of free iron. Notably, blocking ATG5 and ATG7 can only inhibit LC3 lipidation, rather than fulfilling the strict requirements for initiating typical autophagy, which differs from the role of ATG13 and ULK1 in initiating autophagy. Thus, inhibiting NCOA4 alone can partially reduce ferritin degradation and iron release. To effectively suppress iron release and ferroptosis susceptibility, it is necessary to target key autophagy initiation molecules such as ULK1 or ATG13, rather than merely inhibiting ATG5 or ATG7 (which are primarily involved in autophagosome maturation via LC3 lipidation) (12).

Iron excretion mainly relies on membrane iron transporters. Their functions need to work in coordination with ferroxidases such as ceruloplasmin (CP) or heme carrier protein (HEPH) (11). Hepcidin (HAMP) can induce the degradation of ferroportin and thus inhibit iron excretion. Furthermore, poly(rC)-binding protein 1/2 (PCBP1/2) acts as an iron molecular chaperone and participates in intracellular iron transport; notably, the absence of PCBP1 can lead to the accumulation of iron toxicity. Additionally, iron regulatory protein 1/2 (IRP1/2) and AMP-activated protein kinase (AMPK) maintain iron homeostasis by regulating the expression of genes related to iron metabolism and the autophagy process (13). Imbalance in any of the above links may lead to excessive accumulation of Fe^2+^, thereby inducing lipid peroxidation and iron death.

### 2.2 Uncontrolled lipid peroxidation

Lipid peroxidation is a key biochemical process in ferroptosis. Its occurrence depends on three key conditions: a sufficient substrate, free radical initiation, and failed clearance.

Polyunsaturated fatty acids (PUFAs), which serve as the main substrates for lipid peroxidation, require esterification and incorporation into membrane phospholipids—especially polyunsaturated fatty acid-phosphatidylethanolamine (PUFA-PE)—to participate in ferroptosis signaling processes (14). Specifically, Acyl-CoA Synthetase Long-Chain Family Member 4 (ACSL4) activates PUFAs, while Lysophosphatidylcholine Acyltransferase 3 (LPCAT3) catalyzes the synthesis and membrane remodeling of PUFA-PE. Together, these enzymes promote the accumulation of lipid peroxide substrates, facilitating ferroptosis (11).

Fe^2+^ within cells generates hydroxyl radicals (·OH) through the Fenton reaction, thereby initiating the peroxidation of PUFAs (10). Additionally, lipoxygenase (LOXs) may directly facilitate this process. The peroxidation process is negatively regulated by the Xc^-^-GSH-GPX4 axis. The system Xc^-^, composed of solute carrier family 7 member 11 (SLC7A11) and solute carrier family 3 member 2 (SLC3A2), mediates cystine uptake for the synthesis of Glutathione(GSH). Subsequently, glutathione peroxidase 4 (GPX4) utilizes GSH to convert LPO into harmless lipid alcohols (15, 16). Inhibition of this pathway (such as erastin inhibiting SLC7A11 or BECN1 binding to SLC7A11, blocking its function) will lead to a decrease in antioxidant capacity, thereby weakening the antioxidant capacity of cells.

GPX4 is a key enzyme for clearing LPO, and its activity is highly dependent on the level of GSH (17). If GPX4 is directly inhibited or the synthesis of upstream GSH is blocked, it will lead to the accumulation of LPO. In this context, the transcription factor nuclear factor erythrocyte 2-related factor 2 (NRF2) can maintain reduction-oxidation (REDOX) balance by regulating GSH synthase, GPX4, and iron metabolism-related genes (such as HO-1 and ferritin). However, it is essential to note that excessive activation of HO-1 can release Fe^2+^, which in turn promotes oxidation (18). In addition to the mechanisms above, selective autophagy—such as ferritin autophagy, which releases iron, and lipid autophagy, which provides PUFAs—can also further accelerate the lipid peroxidation process.

### 2.3 Antioxidant system imbalance

Cells have several antioxidant protection systems. If these working paths are destroyed, their resistance to ferroptosis will be greatly changed.

The Xc^-^-GSH-GPX4 axis is a classic pathway for intracellular antioxidant defense (19). Impaired function of this pathway significantly increases the susceptibility of cells to ferroptosis.

The FSP1-CoQ10-NAD (P) H pathway is to transform coenzyme Q10 (CoQ10) into its reduced form, panthenol (CoQ10H), by ferroptosis suppressor protein 1 (FSP1), and a process dependent on NAD (P) H. CoQ10H_2_ is a lipid-soluble antioxidant that directly scavenges lipid peroxide free radicals (20), and its effect is independent of GPX4.

The GCH1-BH4 pathway involves GTP cyclohydrolase 1 (GCH1), which catalyzes the synthesis of tetrahydrobiopterin (BH4) (21). BH4 and its metabolites have the ability to directly eliminate free radicals and regulate the expression of genes related to lipid metabolism, thereby reducing the generation of lipid peroxidation substrates.

The transcriptional regulatory network is controlled by the nuclear factor NRF2, which is the primary regulator of our body’s antioxidant response. NRF2 helps cells resist oxidative stress by enhancing many genes that protect cells, such as SLC7A11, GPX4, and GSH synthase (22). Cells resist oxidative stress by countering ROS-induced lipid peroxidation, a key mechanism to prevent ferroptosis. As a core regulator of the oxidative stress-ferroptosis axis, p53 regulates this balance through an environment-dependent biphase pattern: under mild oxidative stress, it mediates peroxidized lipid detoxification by inducing calcium-independent phospholipase A_2_β (iPLA2β), enhancing oxidative stress resistance and maintaining cell survival; activate p21 or regulate NRF2 to enhance antioxidant defense; when oxidative stress intensifies, it inhibits SLC7A11 to disrupt GSH synthesis, thereby weakening the antioxidant stress resistance and triggering ferroptosis to clear damaged cells. This core mediating role of p53, combined with the regulatory participation of NRF2 in antioxidant defense, closely links oxidative stress resistance to ferroptosis outcomes (23). Similarly, the BTB domain and CNC homolog 1 (BACH1) also antagonize NRF2, which promotes ferroptosis by reducing the activity of antioxidant genes, increasing the level of unstable iron, and increasing the expression of long-chain acyl-CoA synthetase 4 (24).

## 3 Biological role of ferroptosis

Ferroptosis is a key regulatory factor in the occurrence, development, and treatment response of digestive tract malignancies. As a strictly regulated cell death process, ferroptosis plays a dual regulatory role in tumor biology, influencing the proliferation, metastasis, and drug resistance-related signaling pathways of tumor cells. On the one hand, ferroptosis can exert anti-cancer effects through mechanisms such as selectively eliminating tumor cells and inhibiting the stemness of tumor stem cells; on the other hand, tumor cells can gain a survival advantage by reshaping the regulatory pathways related to ferroptosis, thereby influencing disease progression and treatment outcomes. This section will clarify the molecular regulatory framework of ferroptosis in digestive tract tumors and systematically expound its multiple roles and mechanisms in tumor biology.

### 3.1 Molecular regulation in digestive tract cancer cells

The initiation and control of ferroptosis depend on complex molecular pathways that involve key proteins in iron homeostasis, non-coding RNA, and various elements within the tumor microenvironment (TME). These components show different expression patterns in different gastrointestinal cancers and are related to the prognosis of the disease.

#### 3.1.1 Regulation of ferroptosis

**Figure 2:**
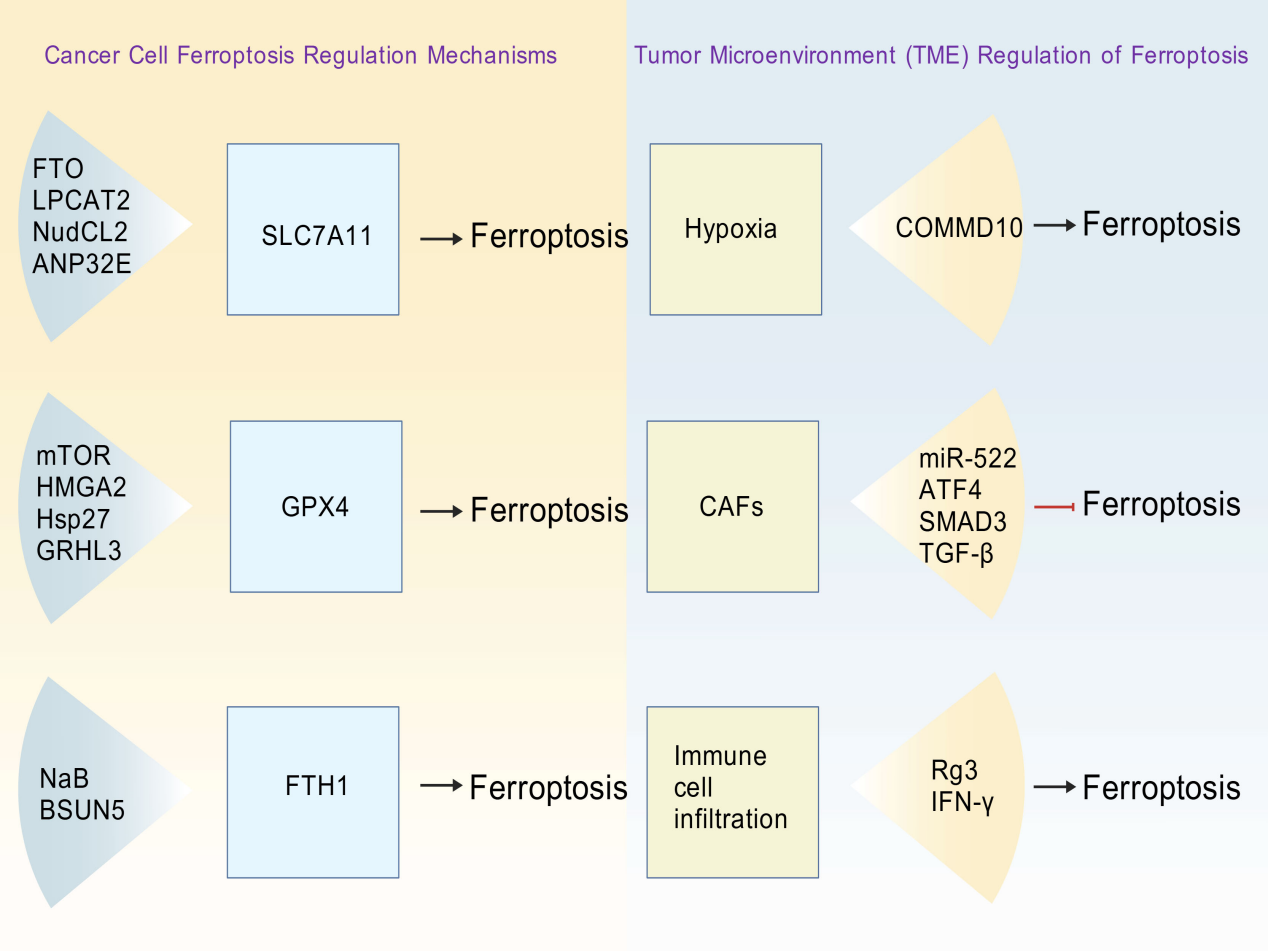
Schematic diagram of iron death regulatory mechanisms in cancer cells and iron death modulation in the tumor microenvironment (TME). The left panel depicts key targets within cancer cells and their corresponding regulatory factors, which influence ferroptosis by modulating core target activity. The right panel illustrates components of the tumor microenvironment—including hypoxia, cancer-associated fibroblasts (CAFs), and immune cell infiltration—that promote or suppress ferroptosis through factors such as COMM domain containing 10 (COMMD10) and microRNA-522 (miR-522). Created with (9).

The state and expression levels of critical regulatory proteins in the cell determine how the cell responds to signals that trigger ferroptosis (**Figure 2**).

Cells have their own protective mechanisms against this type of death, and the key role is played by GPX4. GPX4 eliminates lipid peroxide-induced damage caused by lipid peroxides and protect the integrity of the cell membrane, thereby preventing ferroptosis (17). Because GPX4 is the core regulator of ferroptosis, it is crucial for maintaining cell survival and balance. It has been found that in gastric cancer (GC), a higher level of GPX4 promotes tumor formation by stimulating the mammalian target of rapamycin (mTOR) signaling pathway (25). In pancreatic cancer, high mobility group at-hook 2 (HMGA2) enhances transcription by directly binding to the GPX4 promoter, promoting GPX4 protein synthesis through the mTORC1-4EBP1-S6K pathway. Forming an HMGA2-GPX4 positive feedback loop that confers ferroptosis tolerance (26). Similarly, in esophageal squamous cell carcinoma (ESCC), the upregulation of heat shock protein 27 (Hsp27) increases the expression of GPX4, thereby inhibiting ferroptosis in cancer stem cells (CSCs), and is associated with a poor prognosis for patients (27). R et al. found that in hepatocellular carcinoma (HCC) (28), nuclear-enriched GPX4 inhibits grainyhead-like 3 (GRHL3) transcriptionally, thereby weakening its control over the PTEN/PI3K/AKT pathway and promoting tumor metastasis. Therefore, the expression level of GPX4 in digestive tract tumor tissues is significantly increased, and it confers ferroptosis resistance through multiple mechanisms, becoming a key driver of tumor progression and metastasis.

SLC7A11, as a functional subunit of System xc^-^, can directly affect the sensitivity of GSH synthesis and ferroptosis. Downregulated SLC7A11 expression reduces GSH levels, increasing cellular susceptibility to ferroptosis. Studies have shown that in CRC, N6-methyladenosine (m6A) demethylase fat mass and obesity-associated protein (FTO) can upregulate the expression of SLC7A11 through the M6A-YTHDF2-dependent pathway, thereby protecting CRC cells from ferroptosis and promoting tumorigenesis. In addition, inhibiting FTO or using the novel FTO inhibitor mupirocin can induce ferroptosis in CRC cells, enhance their sensitivity to ferroptosis inducers, such as erastin and RAS selective lethal 3 (RSL3), and inhibit tumor growth (29). In addition, lysophosphatidylcholine acyltransferase 2 (LPCAT2) also induces ferroptosis in CRC cells by inhibiting the nuclear translocation of protein arginine methyltransferase 1 (PRMT1) and suppressing the expression of SLC7A11 (30). In pancreatic cancer (31), low expression of nudc domain-containing protein 2 (NudCL2) is associated with a poor prognosis in patients. Its absence can upregulate SLC7A11, promoting cell migration and invasion, while inhibition of SLC7A11 can reverse this phenotype, indicating that NudCL2 can inhibit epithelial-mesenchymal transition (EMT) by regulating the transcriptional activity of SLC7A11. Highlighting SLC7A11 as a potential target for inhibiting tumor growth.

Delivering iron into the cytoplasm, ferric iron from 1 (FTH1) is the key iron metabolism protein (32). Umathum et al. discovered that in CRC, NaB can induce ferritin autophagy by upregulating NCOA4 and degrading FTH1, thereby promoting intracellular Fe^2+^ accumulation and ferroptosis, which inhibits the growth of transplanted tumors (33). Studies have shown that in Kirsten rat sarcoma viral oncogene homolog (KRAS) mutant pancreatic ductal adenocarcinoma (PDAC), the expression of FTH1 contributes to cell survival and tumorigenesis. This suggests that FTH1 plays a significant role in supporting malignant progression. However, the regulation of miRNA-5000-3p can counteract FTH1’s effects, leading to metabolic disorders and worsening disease progression (34). FTH1 in GC is a key molecule downstream of NSUN5. The NSUN5-FTH1 axis is activated by blocking erastin-induced iron death, promoting SGC7901 cell growth and tumor development *in vivo* (35). These studies reveal that FTH1 has multiple roles in tumor metabolism and cell fate. It relates to both ferroptosis susceptibility and metabolic reprogramming.

In conclusion, GPX4, SLC7A11, FTH1, and other key regulatory proteins form an interconnected network that can not only jointly regulate the ferroptosis process in digestive tract cancer but also serve as potential biomarkers to predict treatment response.

#### 3.1.2 Non-coding RNA regulation of ferroptosis

**Figure 3:**
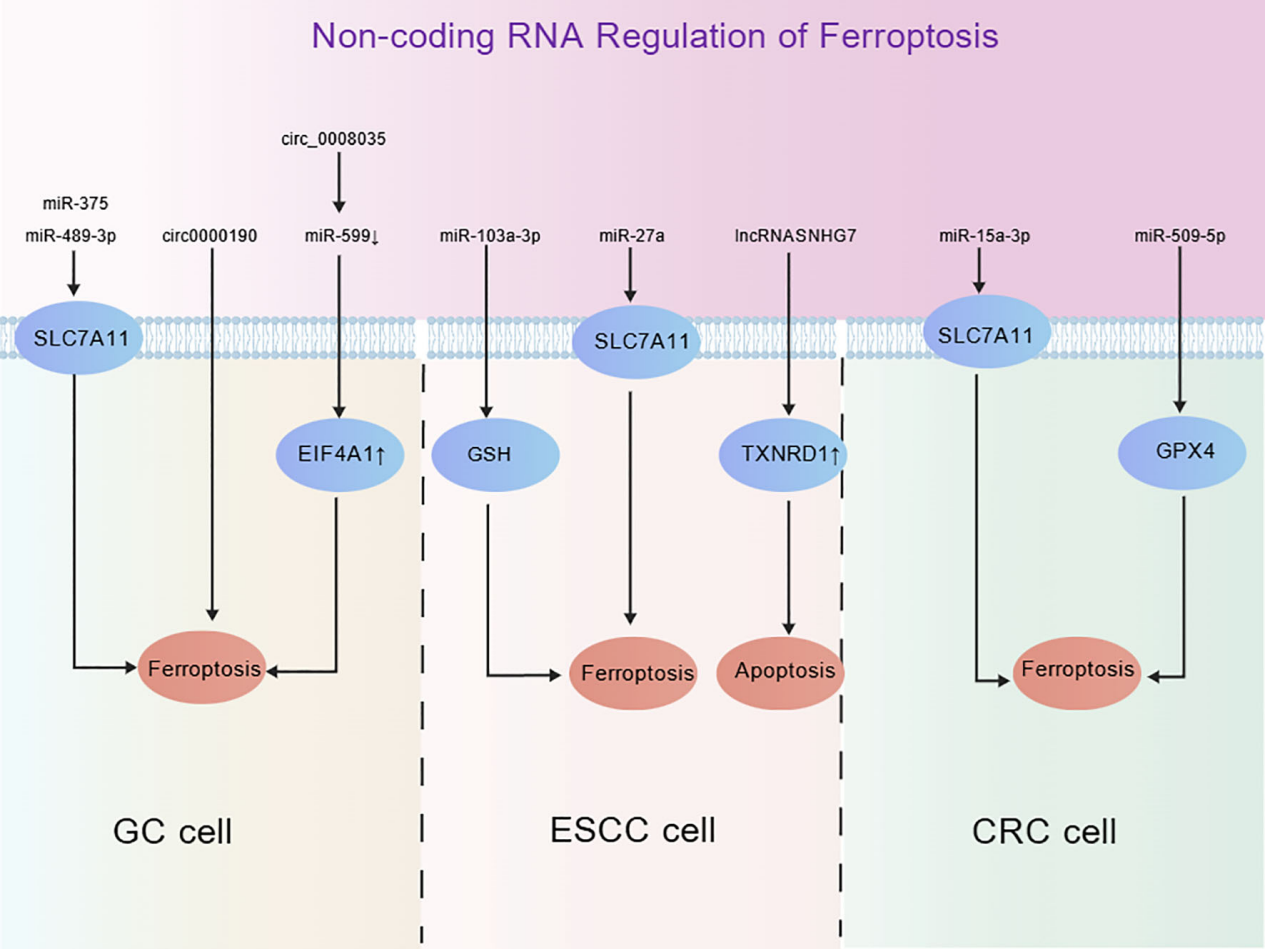
Schematic diagram of non-coding RNA regulatory mechanisms of ferroptosis in gastric cancer (GC), esophageal squamous cell carcinoma (ESCC), and colorectal cancer (CRC) cells. Regulatory pathways governing ferroptosis and apoptosis in various cancer cells are modulated through the action of different non-coding RNAs (such as miRNAs, circular RNAs, and long non-coding RNAs) on target molecules. Created with (9).

Non-coding RNAs (ncRNAs)—including microRNA (miRNA), long non-coding RNA (lncRNA), and circular RNA (circRNA)—play essential roles (36). Play an essential role in regulating the expression of key genes involved in ferroptosis at the epigenetic and post-transcriptional regulatory levels, thereby affecting the sensitivity of digestive tract tumor cells to ferroptosis (**Figure 3**).

As a key post-transcriptional regulator, miRNA has been demonstrated by several studies to have a significant impact on regulating ferroptosis. MiR-103a-3p is an oncogenic miRNA associated with GC development (37); miR-103a-3p influences the ferroptosis process in GC cells by modulating GSH levels. Additionally, the local anesthetic bupivacaine can inhibit the proliferation of GC cells by inducing ferroptosis through the miR-489-3p/SLC7A11 axis. Similarly, miR-375 can mediate the occurrence of Helicobacter pylori-related GC by inhibiting the JAK2-STAT3 signaling pathway (38), and can trigger ferroptosis by targeting SLC7A11 (39). However, notably, in CRC, the expression levels of miR-509-5p and miR-15a-3p are significantly decreased compared with normal colorectal cells. The overexpression of miR-509-5p and miR-15a-3p can promote iron cell apoptosis in CRC cells by targeting SLC7A11 and GPX4, respectively (40) (**Table 1**).

**Table 1:** Non-coding RNA-mediated regulation of ferroptosis.

Non-coding RNA types	Key molecules	Regulatory targets	Effect result
miRNA	miR-103A-3p	GSH	Inhibiting or promoting ferroptosis, GC
	miR-489-3p	SLC7A11	Promotes ferroptosis, GC
	miR-509-5p	SLC7A11/GPX4	Promote ferroptosis,CRC
	miR-15a-3p	SLC7A11/GPX4	Promote ferroptosis,CRC
	miR-375	SLC7A11	Promotes ferroptosis, GC
LncRNA	LncRNA SNHG7	P15/P16	Inhibition of ferroptosis, ESCC
CircRNA	Circ0008035	miR-599/EIF4A1	Inhibits ferroptosis, GC
	Circ0000190		Promote ferroptosis, ESCC
	Circ0120816		Promotes ferroptosis by regulating GSH synthesis, ESCC

LncRNA, which is typically more than 200 nucleotides in length and does not encode proteins, has yielded significant research achievements in the study of various cancers (41). Recently, in the study of ESCC, it was found that the LncRNA SNHG7 was significantly upregulated in ESCC cells and tissues. By regulating the expression of p15 and p16, it could promote the proliferation ability of ESCC cells and inhibit their apoptosis (27) (**Table 1**).

Circular RNA, as an emerging non-coding RNA, is also involved in ferroptosis-related processes (42). Studies have confirmed that in GC cells, the upregulation of circ_0000190 and circ_0008035 expression can inhibit tumor cell proliferation and migration by inducing ferroptosis (43). The latter inhibits apoptosis and ferroptosis of GC cells directly by regulating the miR-599/EIF4A1 axis (44). However, in ESCC, circ_0120816, as a miRNA sponge of miR-1305, can not only promote the development of ESCC but also directly target the key enzyme thioredoxin reductase 1 (TXNRD1) for GSH synthesis, thereby exerting its anti-cancer effect through miR-1305. This indicates that targeting circ_0120816 may regulate the initiation mechanism of ferroptosis by influencing GSH synthesis (45) (**Table 1**).

In conclusion, ncRNA plays a crucial yet complex role in regulating ferroptosis in digestive system tumors. Existing studies have initially revealed the action pathways and mechanisms of some ncRNAs; however, the overall interaction network between ncRNAs and ferroptosis remains far from being fully elucidated. Therefore, in the future, developing more efficient technical platforms to comprehensively depict the ncRNAs’ regulatory network and verify it in combination with clinical samples will be the key direction for technological breakthroughs.

#### 3.1.3 TME regulation of ferroptosis

TME coordinately regulates tumor cell ferroptosis susceptibility through three core mechanisms (46): hypoxia, cancer-associated fibroblasts (CAFs), and immune cell infiltration (**Figure 2**). Under hypoxia, HIF-1α is stabilized by low COMMD10 expression, promoting the transcription of ceruloplasmin and SLC7A11 and inhibiting ferroptosis in HCC. Concurrently, the lncRNA CBSLR reduces cystathionine β-Synthase (CBS) mRNA stability in a YTHDF2-dependent manner, decreasing ACSL4 protein levels and weakening the synthesis of pro-ferroptotic phosphatidylethanolamine, conferring chemotherapy resistance in GC (35). A study demonstrated that CAFs inhibit ferroptosis in pancreatic cancer through two mechanisms: primarily by secreting exosomes with miR-522 that directly target and inhibit arachidonate 15-lipoxygenase (ALOX15); additionally, through the TGF-β/SMAD3/ATF4 signaling pathway, which facilitates cysteine secretion to increase GSH synthesis (47). In the immune microenvironment, the key immune cells, CD8^+^ T cells, secrete interferon-γ (IFN-γ). IFN-γ not only downregulates SLC7A11 but also upregulates ACSL4. Notably, this regulatory pattern—via the apolipoprotein L3 (APOL3)-lactate dehydrogenase A (LDHA) axis—reduces intracellular lactic acid levels and enhances CD8^+^ T cell cytotoxicity, which may eventually lead to their direct triggering of ferroptosis in target tumor cells. Furthermore, studies have shown that Rg3 can further enhance the activity of CD8^+^ T cells by regulating the circFOXP1-miR-4477a-PD-L1 axis and cooperatively induce ferroptosis and apoptosis in gallbladder cancer (GBC) cells (48).

However, the impact of TME does not always promote ferroptosis. For instance, immunosuppressive cell populations, such as M2-type macrophages, can protect tumor cells from the threat of ferroptosis by secreting antioxidants or directly blocking the ferroptosis signaling pathway (49). TME hypoxia, CAF metabolic changes, and complex immune cell interactions collectively determine whether ferroptosis occurs in tumor cells. Understanding the interaction between these molecules has enabled us to discover many new ways to develop new therapies, such as designing treatments for key components in TME.

### 3.2 Biological functions of ferroptosis in tumor biology

Ferroptosis, as a controlled cell death mechanism, has a prominent impact on the life cycle of digestive tract malignancies. On the one hand, it can exert a tumor suppressor effect by directly killing tumor cells; on the other hand, tumor cells may also adapt to treatment by activating or regulating the ferroptosis pathway, thereby gaining a survival advantage and promoting tumor invasion and metastasis.

#### 3.2.1 Suppression of tumorigenesis and metastasis

Ferroptosis can not only directly eliminate tumor cells but also exert its effects by regulating tumor-related pathways and the microenvironment. Therefore, ferroptosis inducers can prevent EMT by directly eliminating epithelial-phenotype cancer cells and inhibiting key EMT transcription factors. They can also reverse the already formed transformation state (50). Thereby effectively limiting local invasion and distant metastasis of tumors.

Studies have shown that the expression level of sirtuin 3 (SIRT3) in GBC tissues is significantly lower than that in adjacent normal tissues (51), and this decline in expression is associated with a poor prognosis for patients. The mechanism of action of SIRT3 lies in its ability to inhibit the AKT signaling pathway. On the one hand, it alleviates the inhibitory effect of AKT on ACSL4, thereby promoting ferroptosis. On the other hand, it also blocks AKT-mediated EMT. Therefore, SIRT3 can ultimately inhibit the growth and metastasis of GBC (51). It is worth noting that HIF-1α also plays a key role in connecting EMT and ferroptosis interactions. Specifically, EMT may increase the sensitivity of cells to ferroptosis by enhancing the accumulation of PUFAs and iron. However, the antioxidant capacity provided by HIF-1α can partially alleviate this situation. This balance between EMT and ferroptosis is not fixed, and it will be affected by factors such as TME and cell heterogeneity. Suggesting that targeting HIF-1α or its regulatory pathways is a viable therapeutic strategy (52).

Clinical studies confirm that inducing ferroptosis significantly impairs CSC self-renewal capacity. As CSCs play critical roles in tumor recurrence, metastasis, and drug resistance, they are key therapeutic targets (53). Ferroptosis induces mitochondrial D-lactate dehydrogenase (LDHD) inactivation via lipid reactive oxygen species (ROS) amplification, leading to D-lactic acid accumulation. D-lactic acid downregulates xCT/GPX4 and increases Fe^2+^ levels, further accelerating ferroptosis. Concurrently, it oxidizes NANOG/OCT4, remodels the epigenome, degrades membrane lipid rafts, and reverses EMT, stripping CSCs of their self-renewal and tumorigenic capabilities (54).

Interestingly, CSCs are both sensitive and resistant to ferroptosis. CSCs exhibit responsiveness to ferroptosis, with their fate determined by the transient equilibrium among three dimensions. These dimensions encompass antioxidant capacity, free iron pool capacity, and membrane polyunsaturated fatty acid content. When two or more dimensions shift toward a pro-oxidative state, such as reduced DHODH activity due to POLQ or E2F4 inhibition, D-lactic acid accumulation triggered by CDK7-YAP-LDHD pathway disruption, or CD8^+^ IFN-γ silencing of SLC7A11, CSCs lose self-renewal capacity and become susceptible to ferroptosis. Conversely, enhancing axial effects through M2 TGF-β, CAF-cysteine, and stearoyl-CoA desaturase 1 (SCD1) upregulation protects CSCs from ROS damage and maintains stem cell properties (55). Previous studies have demonstrated that we can induce CSCs to be more susceptible to ferroptosis by modulating key signaling pathways, including CD44/NRF2, YAP/TAZ, and autophagy (56). Specifically, inducing ferroptosis disrupts the fundamental processes that maintain the characteristics of CSCs. It disrupts redox homeostasis by inhibiting NRF2-mediated antioxidant responses. Inhibit invasion and metastasis characteristics by down-regulating EMT-related transcription factors, and by altering the activity of iron-dependent epigenetic enzymes, which hinders the epigenetic reprogramming essential for CSC self-renewal (57).

Animal studies have shown that ferroptosis is highly effective in inhibiting the metastasis of various digestive tract cancers to distant organs such as the peritoneum and liver. Especially in GC, the down-regulation of NCOA4, the core receptor for ferritin phagocytosis, has been found to be a key driver of peritoneal metastasis. Decreased expression of NCOA4 will block the release of intracellular iron, thereby restraining the Fenton reaction and ROS generation. Due to the weakened ferroptosis, GC cells acquire anti-apoptotic ability and are more prone to peritoneal dissemination (58). These findings suggest that inducing ferroptosis may be a promising therapeutic strategy for preventing and treating metastatic tumors.

#### 3.2.2 Ferroptosis escape mechanisms in tumor progression

**Figure 4:**
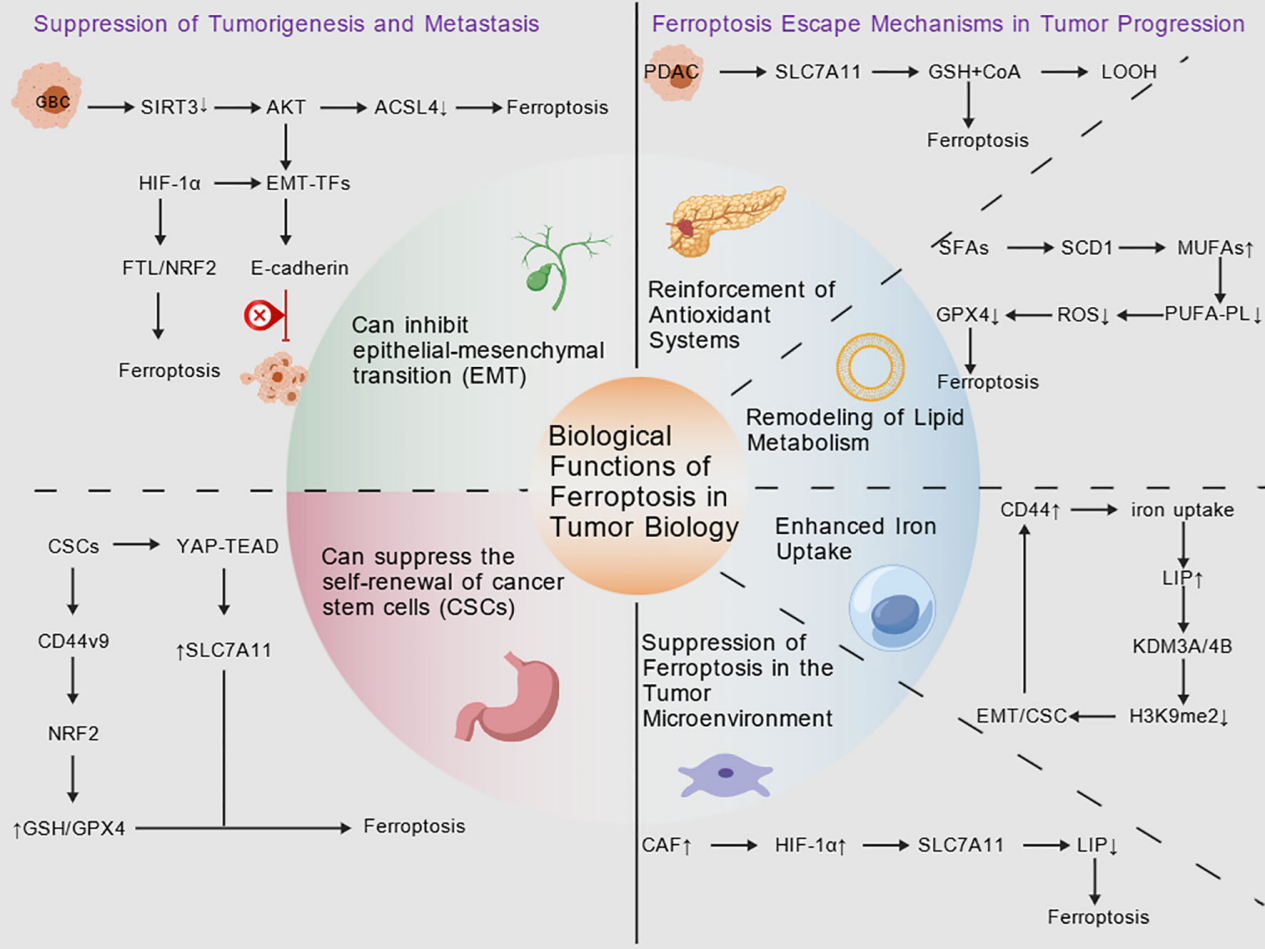
Schematic of ferroptosis’ biological functions in tumor biology. Dual Roles of Ferroptosis in Tumorigenesis and Progression: The left panel illustrates its antitumor effects by inhibiting metastasis, epithelial-mesenchymal transition (EMT), and cancer stem cell (CSC) self-renewal. The right panel depicts how tumors and their microenvironments evade ferroptosis through mechanisms such as enhancing antioxidant systems, reshaping lipid metabolism, upregulating iron uptake, and modulating the microenvironment. Created with (9).

To survive, cancer cells often evade ferroptosis induction through complex escape mechanisms. These escape mechanisms are the key biological basis for tumors to maintain survival, mainly including the upregulation of the antioxidant system, the remodeling of lipid metabolism, the enhancement of iron uptake capacity, and the alteration of the TME (**Figure 4**).

Tumor cells typically upregulate key antioxidant components, such as GPX4 and SLC7A11, to prevent ferroptosis. Merkel et al. believe that mitochondrial-targeted ROS scavengers can inhibit ferroptosis driven by mitochondrial damage by reducing mitochondrial mROS production (59). Specific tumors, such as PDAC, rely on the system xc^-^. This dependence promotes cystine uptake, synchronously synthesizes GSH and CoA, and establishes a synergistic mechanism to resist ferroptosis (60).

Lipid metabolism remodeling—converting saturated fatty acids (SFAs) to monounsaturated fatty acids (MUFAs)—is a key ferroptosis escape mechanism. This conversion is mediated by SCD1. SCD1 can strengthen the cell membrane by producing MUFA. It can reduce the toxicity of ferroptosis inducers in several ways, such as reducing the number of easily oxidized PUFA substrates, reducing the accumulation of ROS, and reducing the expression of GPX4. It has been found that if SCD1 is excessively active, it often indicates a poor prognosis. If SCD1 is inhibited by genetic methods or drugs, the PUFA-dependent ferroptosis process can be restarted (61). Consistent with this, Qin et al. (62) further verified in GC that targeting SCD1 via inhibiting ubiquitin-specific protease 7 (USP7) promotes SCD1 degradation, thereby inducing ferroptosis and suppressing tumor growth. This highlights the congenitally pro-survival effect of SCD1 in digestive tract malignancies, even in different TMEs.

Tumor cells often upregulate the expression of CD44 to enhance their resistance to iron-dependent lipid peroxidation. CD44 is not only important in cell adhesion and migration but also helps cells absorb more iron. Cells start related channels by increasing CD44, allowing iron to enter from outside the cell so that the concentration of free iron in the cell becomes higher (63). Moreover, CD44-mediated iron uptake may drive cancer progression by affecting iron-dependent epigenetic features, such as the demethylation of H3K9me2. This will further enhance the expression of EMT-related genes and ultimately strengthen the stemness characteristics of tumor CSCs.

To resist the attack of ferroptosis inducers and immune cells, tumor cells have developed various mechanisms to inhibit ferroptosis in the TME, among which CAFs provide them with a relatively safe environment. CAFs achieve this protective effect through several ways, including secretion of ferroptosis inhibitors, regulation of iron metabolism, and direct interaction with immunosuppressive cells (64). In addition, some cancer cell membrane-derived vesicles, such as CCM-FSS and CHM-ABI, have also been confirmed to be important inhibitory strategies. Specifically, CCM-FSS refers to the nanoparticle composed of a ferroptosis-sensitizing cascade agent (FSS) encapsulated by a cancer cell membrane (CCM) and is abbreviated as CCM-FSS; CHM-ABI refers to the nanoparticle composed of a dual inhibitor of CXCR4 and NOX4 (ABI) encapsulated by a cancer cell-CAF hybrid membrane (CHM) and is abbreviated as CHM-ABI. These vesicles can not only directly inhibit the ferroptosis of tumor cells themselves through multiple mechanisms but also reprogram CAFs to a quiescent state. Therapeutic strategies based on these vesicles have demonstrated efficacy in orthotopic CRC, colorectal cancer liver metastases (CLM), and humanized immune patient-derived xenograft (PDX) models. This therapy not only effectively inhibits tumor growth but also enhances the anti-tumor immune response, and it shows extremely low toxicity, providing a novel strategy for CRC liver metastases (65).

## 4 Therapeutic strategies targeting iron metabolism

In recent years, several new treatment strategies have emerged. These techniques target malignant tumors of the digestive tract and aim to precisely regulate ferroptosis. The primary strategy is to directly induce ferroptosis in cancer cells. The second approach is a combined strategy, which integrates immunotherapy with ferroptosis treatment. The third technique focuses on treatment sensitivity, making patients more sensitive to radiotherapy. The fourth strategy employs systemic intervention, namely systemic drug treatment. The effects of these four methods are astonishing, generating completely different therapeutic outcomes compared to a single technique (**Table 2**).

**Table 2:** Therapeutic strategies targeting ferroptosis in digestive tract cancer.

Compund/Drug	Cancer type	Target	Function
Simvastatin	GC	SLC7A11/GPX4	Decreases SLC7A11/GPX4 expression and induces ferroptosis in GC cells
DHA DDP	GC	GPX4	Induces ferroptosis in GC cells
L-KYN	GC	GPX4	Leads to NK cell depletion and induces ferroptosis
β-elemene	GC	GPX4	Promotes GPX4 ubiquitination and induces ferroptosis
Shikonin	GC	DLEU1/mTOR/GPX4	Triggers lipid peroxidation and induces ferroptosis
HC-056456	GC	p53/SLC7A11	Disrupts the redox balance and triggers ferroptosis
RSL3 VP CA3	GIST	GPX4	Triggers lipid peroxidation and induces ferroptosis
Imatinib	GIST	STUB1/GPX4	Triggers lipid peroxidation and iron accumulation and induces ferroptosis in GC cells
Ginsenoside Rg3	GBC	The circFOXP1-miR-4477a-PD-L1 axis	Activates CD8^+^ T cell immune function and induces ferroptosis
ISL	GBC	HMOX1 and GPX4	Dual-targets HMOX1 and GPX4 and induces ferroptosis
N6-methyladenosine	ESCC	SOCS6	Suppresses SOCS6 expression, thereby inhibiting ferroptosis in ESCC cells
SAS	ESCC	GPX4	Triggers lipid peroxidation and induces ferroptosis
Gliotoxin	ESCC	SUV39H1	Downregulates SUV39H1 expression and induces ferroptosis
PZM	ESCC	GPX4	Triggers lipid peroxidation and induces ferroptosis
PR-619	CRC	GPX	Induces ferroptosis and promotes CD8^+^ T cell-mediated anti-tumor immunity

### 4.1 Ferroptosis induction

One effective strategy against drug resistance in cancers of the digestive tract is to induce ferroptosis. Using certain ferroptosis inducers and interfering with the intracellular antioxidant system are the two primary approaches to induce ferroptosis. Through the p53/SLC7A11 transcription pathway, the protein acidic nuclear phosphoprotein 32 family member E (ANP32E) regulates both ferroptosis and tumor formation in ESCC. This feature of ANP32E makes it a potentially effective molecular target for eliminating paclitaxel resistance during treatment (66). Statins—clinically used for lipid lowering—also induce ferroptosis. Simvastatin, a statin, decreases interleukin enhancer binding factor 3’s (ILF3) histone H3 lysine 14 (H3K14) acetylation levels while simultaneously stimulating histone deacetylase 6 (HDAC6) expression in GC. ILF3 expression is suppressed as a result of this decline. SLC7A11/GPX4 expression is decreased when ILF3 expression decreases. The DEPTOR and mTOR signaling pathway bear responsibility for this. Ultimately, the entire procedure triggers stomach cancer cells to undergo ferroptosis (67). Since this cascade is confined to the intracellular mevalonate-CoQ10 metabolic pathway and does not intersect with NPC1L1-mediated dietary cholesterol absorption in intestinal epithelium, and since relevant *in vitro* experiments were conducted under standard serum-containing culture conditions without evidence of extracellular lipid deprivation, clinically used doses of simvastatin do not attenuate its ferroptosis-promoting effects by interfering with intestinal lipid absorption. Another study demonstrated that dihydroartemisinin (DHA) and cisplatin (DDP) synergistically inhibit GPX4, inducing ferroptosis in GC cells and enhancing the anti-tumor effect (68). In digestive tract stromal tumors, research by Marine Delvaux et al. showed high sensitivity of gastrointestinal stromal tumors (GIST) to ferroptosis inducers. RSL3, verteporfin (VP, YAP-dependent), and CA3 (YAP-independent) trigger lipid peroxidation and induce ferroptosis by disrupting antioxidant defenses or increasing iron overload. The transferrin receptor (TFRC) was identified as a potential biomarker for predicting ferroptosis sensitivity, suggesting new therapeutic strategies for digestive tract stromal tumors (69). Furthermore, imatinib induces ferroptosis in GIST by upregulating STUB1, which promotes K191 site-specific ubiquitination and degradation of GPX4, thereby disrupting the antioxidant defense, triggering lipid peroxidation, and leading to iron accumulation. The combination of imatinib and RSL3 resulted in increased efficacy (70). Huang et al. demonstrated that the novel multi-kinase inhibitor surufatinib (SUR) in conjunction with photodynamic therapy (PDT) significantly improved anti-tumor effects in cholangiocarcinoma (CCA). The combination treatment triggered ferroptosis in CCA cells through the upregulation of ACSL4 expression, downregulation of GPX4 expression, and an elevation of ROS, LPO, and malondialdehyde (MDA) levels, accompanied by a reduction in GSH levels (71). Furthermore, artemisinin derivatives and photodynamic therapy (PDT) can inhibit CCA by inducing ferroptosis, suggesting novel avenues for CCA treatment (71). In short, although the future development path is still very complicated, the possibility of causing ferroptosis in digestive tract cancer has been confirmed. Utilizing the ferroptosis mechanism presents a novel strategic opportunity for therapeutic intervention and provides a new approach to addressing these intractable malignant tumors.

### 4.2 Immunotherapy

#### 4.2.1 General mechanisms linking ferroptosis to immunogenic cell death

The emergence of immunotherapy represents a significant breakthrough in cancer treatment. It fights cancer by using our own immune system. Immunotherapy leverages the immune system to combat cancer, and its synergy with ferroptosis is supported by their close association with the TME (72). Because ferroptosis is closely related to the TME, the cancer cells killed by ferroptosis not only affect immune cells, but also non-cancer cells related to immune responses are affected in the immune microenvironment. This connection is essential for stopping the spread of cancer. Therefore, it provides robust support for therapeutic modalities in conjunction with immunotherapy. Studies have found that in esophageal cancer, ferroptosis combined with immunotherapy can produce a synergistic effect. CD8^+^ T cells promote ferroptosis in tumors, and inducing ferroptosis can trigger ICD.

Ferroptosis dynamically regulates ICD through three stages (73): early mild lipid peroxidation and mitochondrial DNA (mtDNA) release activate dendritic cells (DCs) and enhance antigen recognition (74); mid-stage massive adenosine triphosphate (ATP) release further recruits T cells, but excessive peroxidation damages the extracellular matrix (ECM) and impedes infiltration (75); and terminal stage membrane rupture massively discharges immunosuppressive factors like TGF-β and adenosine while inducing ferroptosis in T/Natural Killer Cells (NK cells), thereby counteracting antitumor immunity (76). Therefore, a phased precision intervention combining low-dose inducers, ACSL4 agonists, moderate-potency GPX4 inhibitors, and low-dose liproxstatin-1 can maximize the ICD effect and enhance the efficacy of immunotherapy in gastrointestinal tumors. In digestive tract malignancies, ferroptosis activates the DC-cGAS-STING-CD8^+^ T cell axis by triggering classic ICD events. The IFN-γ secreted by CD8^+^ T cells then secondarily upregulates SLC7A11 and ACSL4, further amplifying the sensitivity of cancer cells to ferroptosis. Forming a metabolism-immunity positive feedback loop that deprives CSCs of immune evasion ability and leads to their sustained elimination (77).

#### 4.2.2 Ferroptosis-based strategies to reverse immunotherapy resistance

Targeting ferroptosis may overcome ICB resistance (78). In GC, tumor-associated neutrophils (TANs) play a subtle role. They can induce ferroptosis but do not clear the debris; instead, they release oxidized lipids. TANs release oxidized lipids, impairing anti-tumor immune responses, weakening the immune response that should have attacked the tumor. However, a liposome encapsulating both ferroptosis inhibitor Liproxstatin-1 and modified photosensitizer Icy7, denoted LLI. LLI reshapes the immune landscape through a dual effect—first, it prevents ferroptosis in TANs, and second, it induces ICD in these neutrophils through ceramide accumulation. Notably, this ceramide-mediated ICD reshapes the tumor immune microenvironment, thereby enhancing the efficacy of anti-PD-1 therapy (79). Alternatively, Cui et al. proposed a different mechanism involving L-kyurine (L-KYN). In the immune microenvironment of GC, the indoleamine 2,3-dioxygenase (IDO) enzyme produces L-KYN. They proposed that this L-KYN might prompt NK cells to move towards ferroptosis, but the key lies in avoiding the aromatic hydrocarbon receptor (AHR) pathway. This process will eventually eliminate a large number of NK cells, depleting them to the point where they are no longer able to effectively combat tumors. However, high expression of the ferroptosis protective factor GPX4 shields NK cells from ferroptosis. Thus, genetic engineering to enhance GPX4 expression in NK cells may prolong their survival and activity in the harsh TME, opening new avenues for NK cell-based immunotherapy (76).

#### 4.2.3 The role of ferroptosis-immunotherapy synergy

Consistent with this, in HCC. Zheng et al. revealed a dual mechanism that inhibits ferroptosis and promotes immune escape, thereby facilitating HCC progression. They found that simultaneously targeting the phosphoglycerate Mutase 1 (PGAM1) enzyme could not only activate ferroptosis but also significantly enhance the effect of anti-PD-1 therapy (80). Turning to GBC, Ye et al. focused on a natural compound, ginsenoside Rg3. This substance not only halts iron-induced cell death but also activates CD8^+^ T cells, making them more effective against the cancer. At the same time, Rg3 triggers iron death in GBC cells through a specific molecular pathway (circFOXP1-miR-4477a-PD-L1). It’s like hitting multiple targets at once to fight the tumor (48). In pancreatic cancer research, Li and his team highlighted the pivotal role of the MCP-GPX4/HMGB1 axis in linking ferroptosis to the tumor immune microenvironment. Their research indicates that targeting monocyte chemoattractant protein (MCP) has a dual effect: it can not only induce ferroptosis with “immunogenicity”, but also initiate anti-tumor immune responses by activating M1-type macrophages. The immunogenicity of ferroptosis exhibits stage-dependent characteristics. Its advantage lies in the early exposure of cell membrane transferrin receptors/ATP upon event initiation, activating the dendritic cell-STING-CD8^+^ T cell feedback loop. Its disadvantage involves terminal rupture, releasing transforming growth factor-β, adenosine, and LPO, leading to NK cell/CD8^+^ T cell exhaustion, as well as the recruitment of regulatory T cells. This discovery addresses the issue of ferroptosis inducers potentially increasing immunosuppressive cell numbers, identifying MCP as a promising new target for pancreatic cancer immunotherapy (81).

#### 4.2.4 Bidirectional crosstalk between ferroptosis and TME

Ferroptosis-generated heme/ATP activates the DC-STING-IFN-γ pathway, reversing the TME from a TGF-β/adenosine-driven suppressive state to an inflammatory immune-permissive state by blocking SLC7A11 depletion of Treg/MDSC GSH reserves and eliminating immunosuppressive neutrophils. This enhances the efficacy of ICB in gastrointestinal cancers. These examples demonstrate that there are interesting interactions between them. Ferroptosis seems to regulate the sensitivity of TME by affecting the existence, vitality, and metabolic state of immune cells. Conversely, the characteristics of the immune microenvironment, including cell types and their metabolic states, also seem to affect the sensitivity of cancer cells to ferroptosis. This subtle relationship between ferroptosis and the immune system not only provides valuable scientific insights but also opens up an exciting new avenue for optimizing immunotherapy for digestive tract cancer.

### 4.3 Radiotherapy and ferroptosis

In the past, when we used traditional radiotherapy to deal with cancer, traditional radiotherapy often faces challenges with cancer cell radioresistance. Recently, however, scientists have discovered that combining the regulation of iron metabolism with radiotherapy can overcome this obstacle, making tumor cells more sensitive to radiation. For example, in the case of GC, he and his research team found that β-piperine can inhibit the interaction between OTU domain-containing protein 1 (OTUB1) and GPX4, thereby promoting the ubiquitination and degradation of GPX4 and ultimately triggering the ferroptosis of gastric adenocarcinoma cells with radiation resistance. When this method is used in conjunction with radiotherapy, it shows a significant effect in reversing radiation resistance and effectively inhibiting tumor growth (82). Another study initiated by Wang et al. ingeniously constructed a delivery system based on mesoporous organosilicon nanoparticles (MON), loading them with pyrrolidone (PG). This nanosystem is equipped with an “intelligent” response mechanism and can release PG in a microenvironment rich in GSH. When exposed to X-ray radiation, it can sharply amplify the generation of ROS and simultaneously deplete the GSH reserve. Nearly doubling MDA levels and intensifying lipid peroxidation. As a result, GC cells fell into ferroptosis, accompanied by the induction of DNA damage and mitochondrial dysfunction. The results of animal experiments were equally encouraging. MON@PG, combined with radiotherapy, reduced tumor volume by an astonishing 91.5%, significantly enhancing tumor radiosensitivity and providing a novel radiosensitizing nanoplatform (83). In ESCC, Ma et al. demonstrated that ferroptosis inhibition mediated by m^6^A modification contributes significantly to radioresistance. Methyltransferase-like 3 (METTL3) upregulates m^6^A modification in the 3’ untranslated region (3’ UTR) of suppressor of cytokine signaling 6 (SOCS6) and suppresses SOCS6 expression, thereby inhibiting ferroptosis in ESCC cells (84). In HCC, Chen et al. showed that suppressor of cytokine signaling 2 (SOCS2) promotes ferroptosis and enhances radiosensitivity. SOCS2 specifically recognizes the N-terminal domain of SLC7A11 via its src homology 2 (SH2) domain and, using residues L162/C166 in its BOX domain, binds Elongin B/C to form a complex that recruits ubiquitin molecules, facilitating K48-linked polyubiquitination and degradation of SLC7A11. This reduces cystine uptake, GSH synthesis, and GPX4 levels, inducing ferroptosis and ultimately increasing HCC sensitivity to radiotherapy (85). For CRC, Jin et al.’s report reveals another completely different mechanism. They found that fibronectin 3-like protein 1 (CHI3L1) inhibits ferroptosis by promoting the ubiquitination degradation of p53 and upregulating the expression of SLC7A11. The ultimate result is radioresistance in CRC cells (86). Dai et al. further discovered that in CRC, lncRNA FTX and miR-625-5p jointly regulate the expression of SLC7A11, forming an axis that inhibits ferroptosis and promotes DNA repair. This thereby reduces the sensitivity of tumors to radiotherapy (87). In pancreatic cancer, Zhu et al. depicted a more complex picture. They pointed out that cancer CAF is activated under the activation of the TGF-β/SMAD3/ATF4 pathway and then initiates the transcriptional pathway to secrete cystine. Pancreatic cancer cells take up cystine to synthesize GSH, which in itself inhibits ferroptosis. Meanwhile, GSH also acts as a “scavenger”, eliminating ROS induced by radiotherapy and enhancing the repair of DNA damage. Not to mention, the dense fibrous interstitial structure formed by CAF physically hinders the penetration of radiation. These factors act together, greatly weakening the overall effect of radiotherapy (47). Thus, ferroptosis is a key target for enhancing the radiosensitivity of tumors.

### 4.4 Systemic drugs targeting ferroptosis

Ferroptosis’ potential to overcome drug resistance is supported by growing evidence, with existing drugs and natural compounds effectively targeting tumor cells via this mechanism. Whether it is existing drugs or natural compounds, examples of skillfully utilizing the ferroptosis mechanism to target tumor cells and demonstrate good therapeutic effects are constantly emerging. For example, Valashedi et al. (88) provide us with strong evidence. For example, shikonin’s anti-tumor effect in GC is closely linked to its ferroptosis-inducing activity, and in this process, the DLEU1/mTOR/GPX4 pathway plays a crucial role, as revealed by Wang et al. (89). Meanwhile, the Zhang team, through a drug reutilization screening strategy, unexpectedly discovered a novel ferroptosis inducer—HC-056456. This compound precisely triggers the ferroptosis process in GC cells by disrupting the intracellular REDOX balance and then through the p53/SLC7A11 pathway. It demonstrated significant anti-GC activity *in vitro* and *in vivo*, with favorable drug-like properties, providing a new candidate drug and validating the feasibility of targeting ferroptosis in GC therapy (90). In ESCC, Yin et al. demonstrated that sulfasalazine (SAS) inhibits ESCC cell proliferation by activating ferroptosis (91). Zhang et al. showed that gliotoxin induces ferroptosis in ESCC cells by downregulating SUV39H1 expression (92). He et al. found that pizotifen maleate (PZM), a novel NRF2 inhibitor, suppresses tumor growth by inducing ferroptosis, elucidating an NRF2-targeted therapeutic strategy for ESCC (93). In cholangiocarcinoma, An et al. demonstrated that hypericin-mediated PDT (HY-PDT) induces ferroptosis in CCA cells by inhibiting the AKT/mTORC1/GPX4 axis, downregulating GPX4, decreasing GSH, and increasing ROS and lipid peroxidation products, while also suppressing proliferation, migration, and EMT. These effects were reversible by ferroptosis inhibitors (e.g., Liproxstatin-1) or AKT/mTOR activators (e.g., SC79, MHY1485) (94). In GBC, isoliquiritigenin (ISL), a chalcone derived from licorice, induces ferroptosis in GBC cells by dual-targeting heme oxygenase 1 (HMOX1) and GPX4, consequently inhibiting tumor progression (95). In colon cancer, Wu et al. suggested that the deubiquitinase inhibitor PR-619 may enhance the efficacy of immunotherapy by inducing ferroptosis, thereby promoting CD8^+^ T cell-mediated anti-tumor immunity, indicating a potential combination strategy (96). The research conducted by Huang’s team revealed that NRF2 activation is the core mechanism underlying oxaliplatin resistance in CRC, and this pathway inhibits ferroptosis by upregulating GPX4. Conversely, inhibiting NRF2 will downregulate GPX4 and deplete GSH reserves, thereby intensifying lipid peroxidation, enhancing the chemotherapy-induced ferroptosis process, and simultaneously triggering gasdermin E (GSDME) -mediated pyroptosis. Ultimately, a synergistic “synergistic dual cytotoxic effect” effect on tumor cells was formed (97). It can be seen from this that drugs targeting the iron uptake process, ferroptosis execution, or iron metabolic pathways can not only directly kill tumor cells but also open up new paths for the synergistic effect of chemotherapy and immunotherapy by regulating the cellular iron metabolic state. Currently, relevant research has progressed from the basic experimental stage to the preclinical and early clinical development stage, indicating that this strategy will become a key direction for integrated tumor treatment.

Clinically, ferroptosis confers notable advantages for patients with digestive tract malignancies. It selectively eliminates cancer cells, impedes tumor proliferation and metastasis by targeting EMT and CSCs, and overcomes drug/radiation resistance via remodeling iron metabolism and lipid peroxidation. Moreover, its synergy with immunotherapy, radiotherapy, and systemic drugs enhances therapeutic efficacy while reducing off-target toxicity. As a novel therapeutic axis, ferroptosis-based strategies offer personalized treatment potential through biomarkers such as GPX4 and SLC7A11, promising an improved prognosis for advanced or refractory cases.

## 5 Discussion

Digestive tract malignancies represent a significant global health burden, characterized by high incidence, poor prognosis, and limited treatment options for advanced stages (1, 98). Ferroptosis, an iron-dependent form of regulated cell death driven by lipid peroxidation, has emerged as a pivotal regulator of tumor progression and treatment response, offering new avenues to overcome current limitations, such as drug resistance and low response rates. However, existing research insufficiently explores the TME and its interactions with the immune system, limiting the depth of discussion and requiring targeted expansion.

The TME plays a central role in regulating ferroptosis sensitivity (99). Hypoxic conditions stabilize HIF-1α, upregulating ferroptosis-resistant factors to protect tumor cells from ferroptosis (100). CAFs secrete exosomal miR-522 and promote cysteine secretion, thereby enhancing glutathione synthesis and boosting tumor cells’ resistance to ferroptosis (47). Immune cells within the TME exert dual effects: CD8^+^ T cells secrete IFN-γ to promote ferroptosis (53), whereas M2 macrophages and tumor-associated neutrophils secrete antioxidants or oxidized lipids, both of which inhibit ferroptosis and suppress immune function (76, 79).

The bidirectional crosstalk between ferroptosis and the immune system profoundly influences treatment outcomes, and this complexity offers valuable insights for clinical translation. Ferroptosis-induced ICD can activate the DC-STING-CD8^+^ T cell axis, and IFN-γ secretion further amplifies the effects of ferroptosis, effectively eliminating tumor cells and CSCs and laying the foundation for combination therapy (48). However, issues such as the release of immunosuppressive factors in the late stage of ferroptosis and ferroptosis of immune cells themselves may offset therapeutic benefits (73). This suggests that precise regulation of intervention timing and combination with immunomodulators is necessary to maintain immune dominance. This dual “pro-immune and anti-immune” effect determines that ferroptosis-targeted strategies cannot be applied in isolation but must be deeply integrated with immunotherapy to form a synergistic mechanism (101).

Given these mechanistic features, several key challenges persist in clinical translation. Tumor heterogeneity is further exacerbated by TME diversity, leading to significant variations in ferroptosis sensitivity among different patients, current biomarkers fail to capture TME-immune interactions, hindering patient stratification (77). Additionally, existing ferroptosis inducers suffer from off-target toxicity and poor tumor penetration, which are further aggravated by the physical barriers and metabolic properties of the TME (65).

Future research should integrate ferroptosis with TME remodeling and immunotherapy. Developing TME-responsive targeted delivery systems and combining ferroptosis inducers with immune checkpoint inhibitors or CAF inhibitors can enhance therapeutic efficacy and reduce toxicity (65). Establishing a multi-dimensional biomarker system encompassing ferroptosis-related molecules, TME-immune indicators, and non-coding RNA signatures will enable precise patient stratification.

In summary, ferroptosis holds great potential for the treatment of digestive tract tumors, but its clinical translation relies on in-depth deciphering of the interaction mechanisms between TME, immunity, and ferroptosis. By addressing heterogeneity, developing targeted strategies, and validating predictive biomarkers, we can fully unlock the therapeutic potential of ferroptosis and improve the prognosis of patients with advanced digestive tract malignancies.

## Glossary

3’ UTR: 3’ untranslated region
ACSL4: Acyl-CoA Synthetase Long-Chain Family Member 4
AHR: Aromatic Hydrocarbon Receptor
ALOX15: Arachidonate15-Lipoxygenase
AMPK: AMP-Activated Protein Kinase
ANP32E: Acidic Nuclear Phosphoprotein 32 Family Member E
APOL3: Apolipoprotein L3
ATP: adenosine triphosphate
BACH1: BTB domain and CNC homolog 1
BH4: Tetrahydrobiopterin
CAFs: Cancer-Associated Fibroblasts
CBS: Cystathionine β-Synthase
CCA: Cholangiocarcinoma
CLM: Colorectal Cancer Liver Metastases
CSCs: Cancer Stem Cells
DCs: dendritic cells
DDP: Cisplatin
DHA: Dihydroartemisinin
DMT1: Divalent Metal Transporter 1
ECM: extracellular matrix
EMT: Epithelial-Mesenchymal Transition
ESCC: Esophageal Squamous Cell Carcinoma
FSP1: Ferroptosis Suppressor Protein 1
FTH1: Ferritin Heavy Chain 1
FTL: Ferritin Light Chain
GBC: Gallbladder Cancer
GC: Gastric Cancer
GCH1: GTP Cyclohydrolase 1
GIST: Gastrointestinal Stromal Tumors
GPX4: Glutathione Peroxidase 4
GRHL3: Grainyhead-Like 3
OTUB1: OTU Domain-Containing Protein 1
PDAC: Pancreatic Ductal Adenocarcinoma
PGAM1: Phosphoglycerate Mutase 1
PUFAs: Polyunsaturated Fatty Acids
PZM: Pizotifen Maleate
ROS: Reactive Oxygen Species
RSL3: RAS Selective Lethal 3
SAS: Sulfasalazine
SCD1: Stearoyl-CoA Desaturase 1
SIRT3: Sirtuin 3
GSDME: Gasdermin E
HEPH: Heme Carrier Protein
HIF-1α: Hypoxia-Inducible Factor 1α
HMGA2: High Mobility Group AT-Hook 2
HMOX1: Heme Oxygenase 1
ICB: Immune Checkpoint Inhibitors
ICD: Immunogenic Cell Death
GSH: Glutathione
HCC: Hepatocellular Carcinoma
IDO: Indoleamine 2,3-Dioxygenase
IFN-γ: Interferon-γ
ILF3: Interleukin Enhancer Binding Factor 3
ISL: Isoliquiritigenin
KRAS: Kirsten Rat Sarcoma Viral Oncogene Homolog
LDHA: Lactate Dehydrogenase A
LIP: Labile Iron Pool
LLI: Liproxstatin-1 and modified photosensitizer Icy7
LOXs: Lipoxygenases
LPCAT2: Lysophosphatidylcholine Acyltransferase 2
LPCAT3: Lysophosphatidylcholine Acyltransferase 3
LPO: Lipid Peroxides
m^6^A: N6-Methyladenosine
MDA: Malondialdehyde
METTL3: Methyltransferase-Like 3
MON: Mesoporous Organosilicon Nanoparticles
mtDNA: mitochondrial DNA
mTOR: Mammalian Target of Rapamycin
NCOA4: Nuclear Receptor Coactivator 4
NK Cells: Natural Killer Cells
NRF2: Nuclear Factor Erythrocyte 2-Related Factor 2
NudCL2: NudC Domain Containing 2
SLC3A2: Solute Carrier Family 3 Member 2
SLC7A11: Solute Carrier Family 7 Member 11
SOCS2: Suppressor of Cytokine Signaling 2
SOCS6: Suppressor of Cytokine Signaling 6
TANs: Tumor-Associated Neutrophils
TfR1: Transferrin Receptor 1
TFRC: Transferrin Receptor 1
Tf: Transferrin
TME: Tumor Microenvironment
TXNRD1: Thioredoxin Reductase 1
USP7: ubiquitin-specific protease 7
VP: Verteporfin

## References

[1] Bray F Laversanne M Sung H Ferlay J Siegel RL Soerjomataram I . Global cancer statistics 2022: GLOBOCAN estimates of incidence and mortality worldwide for 36 cancers in 185 countries. CA Cancer J Clin. (2024) 74:229–63. doi: 10.3322/caac.21834, PMID: 38572751

[2] Siegel RL Giaquinto AN Jemal A . Cancer statistic. CA Cancer J Clin. (2024) 74:12–49. doi: 10.3322/caac.21820, PMID: 38230766

[3] Huang J Lucero-Prisno DE Zhang L Xu W Wong SH Ng SC . Updated epidemiology of gastrointestinal cancers in East Asia. Nat Rev Gastroenterol Hepatol. (2023) 20:271–87. doi: 10.1038/s41575-022-00726-3, PMID: 36631716

[4] Liang D Feng Y Zandkarimi F Wang H Zhang Z Kim J . Ferroptosis surveillance independent of GPX4 and differentially regulated by sex hormones. Cell. (2023) 186:2748–2764.e2722. doi: 10.1016/j.cell.2023.05.003, PMID: 37267948 PMC10330611

[5] Dixon SJ Lemberg KM Lamprecht MR Skouta R Zaitsev EM Gleason CE . Ferroptosis: an iron-dependent form of nonapoptotic cell death. Cell. (2012) 149:1060–72. doi: 10.1016/j.cell.2012.03.042, PMID: 22632970 PMC3367386

[6] Xie C Wu N Guo J Ma L Zhang C . The key role of the ferroptosis mechanism in neurological diseases and prospects for targeted therapy. Front Neurosci. (2025) 19:1591417. doi: 10.3389/fnins.2025.1591417, PMID: 40421132 PMC12104224

[7] Brown AR Hirschhorn T Stockwell BR . Ferroptosis-disease perils and therapeutic promise. Science. (2024) 386:848–9. doi: 10.1126/science.adn7030, PMID: 39571009

[8] Lei G Gan B . Exploring Ferroptosis-Inducing Therapies for Cancer Treatment: Challenges and Opportunities. Cancer Res. (2024) 84:961–4. doi: 10.1158/0008-5472.Can-23-4042, PMID: 38558130 PMC10987048

[9] Jiang S Li H Zhang L Mu W Zhang Y Chen T . Generic Diagramming Platform (GDP): a comprehensive database of high-quality biomedical graphics. Nucleic Acids Res. (2025) 53:D1670–6. doi: 10.1093/nar/gkae973, PMID: 39470721 PMC11701665

[10] Stockwell BR . Ferroptosis turns 10: Emerging mechanisms, physiological functions, and therapeutic applications. Cell. (2022) 185:2401–21. doi: 10.1016/j.cell.2022.06.003, PMID: 35803244 PMC9273022

[11] Zhou Q Meng Y Li D Yao L Le J Liu Y . Ferroptosis in cancer: From molecular mechanisms to therapeutic strategies. Signal Transduct. Target. Ther. (2024) 9:55. doi: 10.1038/s41392-024-01769-5, PMID: 38453898 PMC10920854

[12] Hou W Xie Y Song X Sun X Lotze MT Zeh HJ . Autophagy promotes ferroptosis by degradation of ferritin. Autophagy. (2016) 12:1425–8. doi: 10.1080/15548627.2016.1187366, PMID: 27245739 PMC4968231

[13] Singh M Arora HL Naik R Joshi S Sonawane K Sharma NK . Ferroptosis in Cancer: Mechanism and Therapeutic Potential. Int J Mol Sci. (2025) 26:3852. doi: 10.3390/ijms26083852, PMID: 40332483 PMC12028135

[14] Samovich SN Mikulska-Ruminska K Dar HH Tyurina YY Tyurin VA Souryavong AB . Strikingly High Activity of 15-Lipoxygenase Towards Di-Polyunsaturated Arachidonoyl/Adrenoyl-Phosphatidylethanolamines Generates Peroxidation Signals of Ferroptotic Cell Death. Angew. Chem Int Ed. Engl. (2024) 63:e202314710. doi: 10.1002/anie.202314710, PMID: 38230815 PMC11068323

[15] Li FJ Long HZ Zhou ZW Luo HY Xu SG Gao LC . System X(c) (-)/GSH/GPX4 axis: An important antioxidant system for the ferroptosis in drug-resistant solid tumor therapy. Front Pharmacol. (2022) 13:910292. doi: 10.3389/fphar.2022.910292, PMID: 36105219 PMC9465090

[16] Liang D Minikes AM Jiang X . Ferroptosis at the intersection of lipid metabolism and cellular signaling. Mol Cell. (2022) 82:2215–27. doi: 10.1016/j.molcel.2022.03.022, PMID: 35390277 PMC9233073

[17] Sun Y Chen P Zhai B Zhang M Xiang Y Fang J . The emerging role of ferroptosis in inflammation. BioMed Pharmacother. (2020) 127:110108. doi: 10.1016/j.biopha.2020.110108, PMID: 32234642

[18] Xie Y Kang R Klionsky DJ Tang D . GPX4 in cell death, autophagy, and disease. Autophagy. (2023) 19:2621–38. doi: 10.1080/15548627.2023.2218764, PMID: 37272058 PMC10472888

[19] Koppula P Zhuang L Gan B . Cystine transporter SLC7A11/xCT in cancer: ferroptosis, nutrient dependency, and cancer therapy. Protein Cell. (2021) 12:599–620. doi: 10.1007/s13238-020-00789-5, PMID: 33000412 PMC8310547

[20] Zhou J Li Y Xi C Pan W Yu Q Wu Z . Apigenin Alleviates Fumonisin B1-Induced Hepatotoxicity by Suppressing Ferroptosis through the Nrf2/FSP1 Pathway. J Agric Food Chem. (2025) 73:26999–7011. doi: 10.1021/acs.jafc.5c08870, PMID: 41073359

[21] Liu Y Lu S Wu LL Yang L Yang L Wang J . The diversified role of mitochondria in ferroptosis in cancer. Cell Death Dis. (2023) 14:519. doi: 10.1038/s41419-023-06045-y, PMID: 37580393 PMC10425449

[22] Rojo De La Vega M Chapman E Zhang DD . NRF2 and the Hallmarks of Cancer. Cancer Cell. (2018) 34:21–43. doi: 10.1016/j.ccell.2018.03.022, PMID: 29731393 PMC6039250

[23] Chen D Chu B Yang X Liu Z Jin Y Kon N . iPLA2β-mediated lipid detoxification controls p53-driven ferroptosis independent of GPX4. Nat Commun. (2021) 12:3644. doi: 10.1038/s41467-021-23902-6, PMID: 34131139 PMC8206155

[24] Zhao L Kang M Liu X Wang Z Wang Y Chen H . UBR7 inhibits HCC tumorigenesis by targeting Keap1/Nrf2/Bach1/HK2 and glycolysis. J Exp Clin Cancer Res. (2022) 41:330. doi: 10.1186/s13046-022-02528-6, PMID: 36419136 PMC9686014

[25] Chen X Yu C Kang R Kroemer G Tang D . Cellular degradation systems in ferroptosis. Cell Death Differ. (2021) 28:1135–48. doi: 10.1038/s41418-020-00728-1, PMID: 33462411 PMC8027807

[26] Luo Z Zheng Q Ye S Li Y Chen J Fan C . HMGA2 alleviates ferroptosis by promoting GPX4 expression in pancreatic cancer cells. Cell Death Dis. (2024) 15:220. doi: 10.1038/s41419-024-06592-y, PMID: 38493165 PMC10944463

[27] Zhang H Zhang J Luan S Liu Z Li X Liu B . Unraveling the Complexity of Regulated Cell Death in Esophageal Cancer: from Underlying Mechanisms to Targeted Therapeutics. Int J Biol Sci. (2023) 19:3831–68. doi: 10.7150/ijbs.85753, PMID: 37564206 PMC10411468

[28] Pan R Zhao Z Xu D Li C Xia Q . GPX4 transcriptionally promotes liver cancer metastasis via GRHL3/PTEN/PI3K/AKT axis. Trans Res.: J Lab Clin Med. (2024) 271:79–92. doi: 10.1016/j.trsl.2024.05.007, PMID: 38797432

[29] Qiao Y Su M Zhao H Liu H Wang C Dai X . Targeting FTO induces colorectal cancer ferroptotic cell death by decreasing SLC7A11/GPX4 expression. J Exp Clin Cancer Res. (2024) 43:108. doi: 10.1186/s13046-024-03032-9, PMID: 38600610 PMC11005233

[30] Wang X Liu P An Y Hu Y Qiao H Miao H . RBMS2 mediates SLC7A11 transcription-translation to regulate ferroptosis in colorectal cancer. Free Radical Biol Med. (2025) 240:504–13. doi: 10.1016/j.freeradbiomed.2025.08.051, PMID: 40854446

[31] Feng J Yang M Lin R Shang K Sun M Guo Z . NudCL2 suppresses pancreatic cancer progression by inhibiting SLC7A11-mediated EMT and metastasis. Exp Cell Res. (2025) 452:114730. doi: 10.1016/j.yexcr.2025.114730, PMID: 40907782

[32] Umathum V Weber A Amsel D Alexopoulos I Becker C Roth A . Distribution of ferritin complex in the adult brain and altered composition in neuroferritinopathy due to a novel variant in the ferritin heavy chain gene FTH1 (c.409_410del; p.H137Lfs*4). Brain Pathol. (2024) 34:e13176. doi: 10.1111/bpa.13176, PMID: 37265023 PMC10711253

[33] Liu L Liu Y Zhou X He H Chen N Qin Y . Sodium butyrate induces ferroptosis in colorectal cancer cells by promoting NCOA4-FTH1-mediated ferritinophagy. Int Immunopharmacol. (2025) 163:115188. doi: 10.1016/j.intimp.2025.115188, PMID: 40652583

[34] JM P YH S CS F HH C YK Q LL C . Crosstalk between FTH1 and PYCR1 dysregulates proline metabolism and mediates cell growth in KRAS-mutant pancreatic cancer cells. Exp Mol Med. (2024) 56:2065–81. doi: 10.1038/s12276-024-01300-4, PMID: 39294443 PMC11447051

[35] Yang H Hu Y Weng M Liu X Wan P Hu Y . Hypoxia inducible lncRNA-CBSLR modulates ferroptosis through m6A-YTHDF2-dependent modulation of CBS in gastric cancer. J Adv. Res. (2022) 37:91–106. doi: 10.1016/j.jare.2021.10.001, PMID: 35499052 PMC9039740

[36] Zheng Y Liu L Shukla GC . A comprehensive review of web-based non-coding RNA resources for cancer research. Cancer Lett. (2017) 407:1–8. doi: 10.1016/j.canlet.2017.08.015, PMID: 28823961

[37] Jin W Liu J Yang J Feng Z Feng Z Huang N . Identification of a key ceRNA network associated with ferroptosis in gastric cancer. Sci Rep. (2022) 12:20088. doi: 10.1038/s41598-022-24402-3, PMID: 36418919 PMC9684404

[38] Ni H Qin H Sun C Liu Y Ruan G Guo Q . MiR-375 reduces the stemness of gastric cancer cells through triggering ferroptosis. Stem Cell Res Ther. (2021) 12:325. doi: 10.1186/s13287-021-02394-7, PMID: 34090492 PMC8180146

[39] Liu Y Miao R Xia J Zhou Y Yao J Shao S . Infection of Helicobacter pylori contributes to the progression of gastric cancer through ferroptosis. Cell Death Discov. (2024) 10:485. doi: 10.1038/s41420-024-02253-3, PMID: 39622791 PMC11612470

[40] Yang G Qian B He L Zhang C Wang J Qian X . Application prospects of ferroptosis in colorectal cancer. Cancer Cell Int. (2025) 25:59. doi: 10.1186/s12935-025-03641-0, PMID: 39984914 PMC11846473

[41] Tan YT Lin JF Li T Li JJ Xu RH Ju HQ . LncRNA-mediated posttranslational modifications and reprogramming of energy metabolism in cancer. Cancer Commun (Lond). (2021) 41:109–20. doi: 10.1002/cac2.12108, PMID: 33119215 PMC7896749

[42] Wang W Sun L Huang MT Quan Y Jiang T Miao Z . Regulatory circular RNAs in viral diseases: applications in diagnosis and therapy. RNA Biol. (2023) 20:847–58. doi: 10.1080/15476286.2023.2272118, PMID: 37882652 PMC10730172

[43] Wang GJ Yu TY Li YR Liu YJ Deng BB . Circ_0000190 suppresses gastric cancer progression potentially via inhibiting miR-1252/PAK3 pathway. Cancer Cell Int. (2020) 20:351. doi: 10.1186/s12935-020-01422-5, PMID: 32742198 PMC7391524

[44] Li C Tian Y Liang Y Li Q . Circ_0008035 contributes to cell proliferation and inhibits apoptosis and ferroptosis in gastric cancer via miR-599/EIF4A1 axis. Cancer Cell Int. (2020) 20:84. doi: 10.1186/s12935-020-01168-0, PMID: 32190008 PMC7076943

[45] Li X Song L Wang B Tao C Shi L Xu M . Circ0120816 acts as an oncogene of esophageal squamous cell carcinoma by inhibiting miR-1305 and releasing TXNRD1. Cancer Cell Int. (2020) 20:526. doi: 10.1186/s12935-020-01617-w, PMID: 33292234 PMC7597039

[46] He X Guan XY Li Y . Clinical significance of the tumor microenvironment on immune tolerance in gastric cancer. Front Immunol. (2025) 16:1532605. doi: 10.3389/fimmu.2025.1532605, PMID: 40028336 PMC11868122

[47] Zhu Y Fang S Fan B Xu K Xu L Wang L . Cancer-associated fibroblasts reprogram cysteine metabolism to increase tumor resistance to ferroptosis in pancreatic cancer. Theranostics. (2024) 14:1683–700. doi: 10.7150/thno.89805, PMID: 38389839 PMC10879865

[48] Ye Z Ding J Huang J Hu Z Jin F Wu K . Ginsenoside Rg3 activates the immune function of CD8+ T cells via circFOXP1-miR-4477a-PD-L1 axis to induce ferroptosis in gallbladder cancer. Arch Pharm Res. (2024) 47:793–811. doi: 10.1007/s12272-024-01516-y, PMID: 39466543

[49] Huang J Pan H Sun J Wu J Xuan Q Wang J . TMEM147 aggravates the progression of HCC by modulating cholesterol homeostasis, suppressing ferroptosis, and promoting the M2 polarization of tumor-associated macrophages. J Exp Clin Cancer Res. (2023) 42:286. doi: 10.1186/s13046-023-02865-0, PMID: 37891677 PMC10612308

[50] Zhang H Chen N Ding C Zhang H Liu D Liu S . Ferroptosis and EMT resistance in cancer: a comprehensive review of the interplay. Front Oncol. (2024) 14:1344290. doi: 10.3389/fonc.2024.1344290, PMID: 38469234 PMC10926930

[51] Liu L Li Y Cao D Qiu S Li Y Jiang C . SIRT3 inhibits gallbladder cancer by induction of AKT-dependent ferroptosis and blockade of epithelial-mesenchymal transition. Cancer Lett. (2021) 510:93–104. doi: 10.1016/j.canlet.2021.04.007, PMID: 33872694

[52] Shen Z Yu N Zhang Y Jia M Sun Y Li Y . The potential roles of HIF-1α in epithelial-mesenchymal transition and ferroptosis in tumor cells. Cell Signal. (2024) 122:111345. doi: 10.1016/j.cellsig.2024.111345, PMID: 39134249

[53] Wang H Zhang Z Ruan S Yan Q Chen Y Cui J . Regulation of iron metabolism and ferroptosis in cancer stem cells. Front Oncol. (2023) 13:1251561. doi: 10.3389/fonc.2023.1251561, PMID: 37736551 PMC10509481

[54] Lv M Gong Y Liu X Wang Y Wu Q Chen J . CDK7-YAP-LDHD axis promotes D-lactate elimination and ferroptosis defense to support cancer stem cell-like properties. Signal Transduct. Target. Ther. (2023) 8:302. doi: 10.1038/s41392-023-01555-9, PMID: 37582812 PMC10427695

[55] Peng Y Zheng W Chen Y Lei X Yang Z Yang Y . POLQ inhibition attenuates the stemness and ferroptosis resistance in gastric cancer cells via downregulation of dihydroorotate dehydrogenase. Cell Death Dis. (2024) 15:248. doi: 10.1038/s41419-024-06618-5, PMID: 38575587 PMC10995193

[56] Elgendy SM Alyammahi SK Alhamad DW Abdin SM Omar HA . Ferroptosis: An emerging approach for targeting cancer stem cells and drug resistance. Crit Rev Oncol Hematol. (2020) 155:103095. doi: 10.1016/j.critrevonc.2020.103095, PMID: 32927333

[57] Yu R Hang Y Tsai HI Wang D Zhu H . Iron metabolism: backfire of cancer cell stemness and therapeutic modalities. Cancer Cell Int. (2024) 24:157. doi: 10.1186/s12935-024-03329-x, PMID: 38704599 PMC11070091

[58] Huang S Ji P Xu P Liu K Ge H Yan Z . PLAGL2-STAU1-NCOA4 axis enhances gastric cancer peritoneal metastasis by resisting ferroptosis via ferritinophagy. Apoptosis. (2025) 30:1058–75. doi: 10.1007/s10495-025-02083-3, PMID: 39987411

[59] Merkel M Goebel B Boll M Adhikari A Maurer V Steinhilber D . Mitochondrial Reactive Oxygen Species Formation Determines ACSL4/LPCAT2-Mediated Ferroptosis. Antiox. (Basel). (2023) 12:1590. doi: 10.3390/antiox12081590, PMID: 37627584 PMC10451816

[60] Badgley MA Kremer DM Maurer HC DelGiorno KE Lee HJ Purohit V . Cysteine depletion induces pancreatic tumor ferroptosis in mice. Science. (2020) 368:85–9. doi: 10.1126/science.aaw9872, PMID: 32241947 PMC7681911

[61] Guo Z Huo X Li X Jiang C Xue L . Advances in regulation and function of stearoyl-CoA desaturase 1 in cancer, from bench to bed. Sci China Life Sci. (2023) 66:2773–85. doi: 10.1007/s11427-023-2352-9, PMID: 37450239

[62] Guan X Wang Y Yu W Wei Y Lu Y Dai E . Blocking Ubiquitin-Specific Protease 7 Induces Ferroptosis in Gastric Cancer via Targeting Stearoyl-CoA Desaturase. Adv Sci (Weinh). (2024) 11:e2307899. doi: 10.1002/advs.202307899, PMID: 38460164 PMC11095140

[63] Ando T Yamasaki J Saya H Nagano O . CD44: a key regulator of iron metabolism, redox balance, and therapeutic resistance in cancer stem cells. Stem Cells. (2025) 43:sxaf024. doi: 10.1093/stmcls/sxaf024, PMID: 40259468 PMC12126136

[64] Chen X Kang R Kroemer G Tang D . Ferroptosis in infection, inflammation, and immunity. J Exp Med. (2021) 218:e20210518. doi: 10.1084/jem.20210518, PMID: 33978684 PMC8126980

[65] Wu Z Lin X Ying Y Fan G Shi J Zheng X . A dual-targeting strategy to inhibit colorectal cancer liver metastasis via tumor cell ferroptosis and cancer-associated fibroblast reprogramming. Bioact. Mater. (2025) 52:73–91. doi: 10.1016/j.bioactmat.2025.05.025, PMID: 40530415 PMC12173058

[66] Sun LY Ke SB Li BX Chen FS Huang ZQ Li L . ANP32E promotes esophageal cancer progression and paclitaxel resistance via P53/SLC7A11 axis-regulated ferroptosis. Int Immunopharmacol. (2025) 144:113436. doi: 10.1016/j.intimp.2024.113436, PMID: 39566382

[67] Sun D Cui X Yang W Wei M Yan Z Zhang M . Simvastatin inhibits PD-L1 via ILF3 to induce ferroptosis in gastric cancer cells. Cell Death Dis. (2025) 16:208. doi: 10.1038/s41419-025-07562-8, PMID: 40140647 PMC11947124

[68] Wang H Lu C Zhou H Zhao X Huang C Cheng Z . Synergistic effects of dihydroartemisinin and cisplatin on inducing ferroptosis in gastric cancer through GPX4 inhibition. Gastric. Cancer. (2025) 28:187–210. doi: 10.1007/s10120-024-01574-7, PMID: 39733394

[69] Delvaux M Hagué P Craciun L Wozniak A Demetter P Schöffski P . Ferroptosis Induction and YAP Inhibition as New Therapeutic Targets in Gastrointestinal Stromal Tumors (GISTs). Cancers (Basel). (2022) 14:5050. doi: 10.3390/cancers14205050, PMID: 36291834 PMC9599726

[70] Sun X Zhang Q Lin X Shu P Gao X Shen K . Imatinib induces ferroptosis in gastrointestinal stromal tumors by promoting STUB1-mediated GPX4 ubiquitination. Cell Death Dis. (2023) 14:839. doi: 10.1038/s41419-023-06300-2, PMID: 38110356 PMC10728200

[71] Huang YP Wang YX Zhou H Liu ZT Zhang ZJ Xiong L . Surufatinib combined with photodynamic therapy induces ferroptosis to inhibit cholangiocarcinoma *in vitro* and in tumor models. Front Pharmacol. (2024) 15:1288255. doi: 10.3389/fphar.2024.1288255, PMID: 38645554 PMC11027741

[72] Riley RS June CH Langer R Mitchell MJ . Delivery technologies for cancer immunotherapy. Nat Rev Drug Discov. (2019) 18:175–96. doi: 10.1038/s41573-018-0006-z, PMID: 30622344 PMC6410566

[73] Wiernicki B Maschalidi S Pinney J Adjemian S Vanden Berghe T Ravichandran KS . Cancer cells dying from ferroptosis impede dendritic cell-mediated anti-tumor immunity. Nat Commun. (2022) 13:3676. doi: 10.1038/s41467-022-31218-2, PMID: 35760796 PMC9237053

[74] Xia W Lv Y Zou Y Kang Z Li Z Tian J . The role of ferroptosis in colorectal cancer and its potential synergy with immunotherapy. Front Immunol. (2024) 15:1526749. doi: 10.3389/fimmu.2024.1526749, PMID: 39850905 PMC11754392

[75] Liu Y Niu R Zhao H Wang Y Song S Zhang H . Single-Site Nanozymes with a Highly Conjugated Coordination Structure for Antitumor Immunotherapy via Cuproptosis and Cascade-Enhanced T Lymphocyte Activity. J Am Chem Soc. (2024) 146:3675–88. doi: 10.1021/jacs.3c08622, PMID: 38305736

[76] Cui JX Xu XH He T Liu JJ Xie TY Tian W . L-kynurenine induces NK cell loss in gastric cancer microenvironment via promoting ferroptosis. J Exp Clin Cancer Res. (2023) 42:52. doi: 10.1186/s13046-023-02629-w, PMID: 36855135 PMC9976385

[77] Wang W Green M Choi JE Gijón M Kennedy PD Johnson JK . CD8(+) T cells regulate tumour ferroptosis during cancer immunotherapy. Nature. (2019) 569:270–4. doi: 10.1038/s41586-019-1170-y, PMID: 31043744 PMC6533917

[78] Fan X Fan YT Zeng H Dong XQ Lu M Zhang ZY . Role of ferroptosis in esophageal cancer and corresponding immunotherapy. World J Gastrointest. Oncol. (2023) 15:1105–18. doi: 10.4251/wjgo.v15.i7.1105, PMID: 37546564 PMC10401468

[79] Zhu X Zheng W Wang X Li Z Shen X Chen Q . Enhanced Photodynamic Therapy Synergizing with Inhibition of Tumor Neutrophil Ferroptosis Boosts Anti-PD-1 Therapy of Gastric Cancer. Adv Sci (Weinh). (2024) 11:e2307870. doi: 10.1002/advs.202307870, PMID: 38233204 PMC10966534

[80] Zheng Y Wang Y Lu Z Wan J Jiang L Song D . PGAM1 Inhibition Promotes HCC Ferroptosis and Synergizes with Anti-PD-1 Immunotherapy. Adv Sci (Weinh). (2023) 10:e2301928. doi: 10.1002/advs.202301928, PMID: 37705495 PMC10582428

[81] Li G Liao C Chen J Wang Z Zhu S Lai J . Targeting the MCP-GPX4/HMGB1 Axis for Effectively Triggering Immunogenic Ferroptosis in Pancreatic Ductal Adenocarcinoma. Adv Sci (Weinh). (2024) 11:e2308208. doi: 10.1002/advs.202308208, PMID: 38593415 PMC11151063

[82] He J Li M Bao J Peng Y Xue W Chen J . β-Elemene promotes ferroptosis and reverses radioresistance in gastric cancer by inhibiting the OTUB1-GPX4 interaction. Front Pharmacol. (2024) 15:1469180. doi: 10.3389/fphar.2024.1469180, PMID: 39484165 PMC11524901

[83] Wang H Niu H Luo X Zhu N Xiang J He Y . Radiosensitizing effects of pyrogallol-loaded mesoporous or-ganosilica nanoparticles on gastric cancer by amplified ferroptosis. Front Bioeng. Biotechnol. (2023) 11:1171450. doi: 10.3389/fbioe.2023.1171450, PMID: 37143600 PMC10151506

[84] Ma R Zhao L . The Role and Mechanism of Ferroptosis Mediated by METTL3-m6A Modification in Regulating Radioresistance of Esophageal Cancer. Int J Radiat Oncol. Biol. Phys. (2023) 117:e248–9. doi: 10.1016/j.ijrobp.2023.06.1188

[85] Chen Q Zheng W Guan J Liu H Dan Y Zhu L . SOCS2-enhanced ubiquitination of SLC7A11 promotes ferroptosis and radiosensitization in hepatocellular carcinoma. Cell Death Differ. (2023) 30:137–51. doi: 10.1038/s41418-022-01051-7, PMID: 35995846 PMC9883449

[86] Jin M Liu H Zheng Z Fang S Xi Y Liu K . CHI3L1 mediates radiation resistance in colorectal cancer by inhibiting ferroptosis via the p53/SLC7A11 pathway. J Transl Med. (2025) 23:357. doi: 10.1186/s12967-025-06378-6, PMID: 40119400 PMC11929242

[87] Dai Q Qu TY Yang JL Leng J Fang L Zhu QQ . LncRNA FTX promotes colorectal cancer radioresistance through disturbing redox balance and inhibiting ferroptosis via miR-625-5p/SCL7A11 axis. World J Gastroenterol. (2025) 31:104305. doi: 10.3748/wjg.v31.i16.104305, PMID: 40308806 PMC12038530

[88] Valashedi MR Nikoo A Najafi-Ghalehlou N Tomita K Kuwahara Y Sato T . Pharmacological Targeting of Ferroptosis in Cancer Treatment. Curr Cancer Drug Targets. (2022) 22:108–25. doi: 10.2174/1568009621666211202091523, PMID: 34856903

[89] Wang Y Xu M Liu C Wang X Zhang X Sheng W . Induction of Ferroptosis by Shikonin in Gastric Cancer via the DLEU1/mTOR/GPX4 Axis. Cell Biol Int. (2025) 49:757–71. doi: 10.1002/cbin.70018, PMID: 40126008

[90] Zhang J Gao M Niu Y Sun J . Identification of a Novel Ferroptosis Inducer for Gastric Cancer Treatment Using Drug Repurposing Strategy. Front Mol Biosci. (2022) 9:860525. doi: 10.3389/fmolb.2022.860525, PMID: 35860356 PMC9289365

[91] Yin LB Li ZW Wang JL Wang L Hou L Hu SY . Sulfasalazine inhibits esophageal cancer cell proliferation by mediating ferroptosis. Chem Biol Drug Des. (2023) 102:730–7. doi: 10.1111/cbdd.14281, PMID: 37291716

[92] Zhang S Guo J Zhang H Tong L Zhang L . Gliotoxin Induced Ferroptosis by Downregulating SUV39H1 Expression in Esophageal Cancer Cells. Recent Pat Anticancer Drug Discov. (2023) 18:397–407. doi: 10.2174/1574892817666220905114120, PMID: 36065932

[93] He X Zhou Y Chen W Zhao X Duan L Zhou H . Repurposed pizotifen malate targeting NRF2 exhibits anti-tumor activity through inducing ferroptosis in esophageal squamous cell carcinoma. Oncogene. (2023) 42:1209–23. doi: 10.1038/s41388-023-02636-3, PMID: 36841865

[94] An W Zhang K Li G Zheng S Cao Y Liu J . Hypericin mediated photodynamic therapy induces ferroptosis via inhibiting the AKT/mTORC1/GPX4 axis in cholangiocarcinoma. Transl Oncol. (2025) 52:102234. doi: 10.1016/j.tranon.2024.102234, PMID: 39674093 PMC11700288

[95] Tie Y Chen M Zhang S . Insights into the molecular mechanisms and therapeutic implications of interleukin-6 for inflammatory bowel disease. Chin Med J (Engl). (2023) 136:2143–6. doi: 10.1097/cm9.0000000000002792, PMID: 37415535 PMC10508555

[96] Wu J Liu C Wang T Liu H Wei B . Deubiquitinase inhibitor PR-619 potentiates colon cancer immunotherapy by inducing ferroptosis. Immunology. (2023) 170:439–51. doi: 10.1111/imm.13683, PMID: 37526037

[97] Huang Y Yang W Yang L Wang T Li C Yu J . Nrf2 inhibition increases sensitivity to chemotherapy of colorectal cancer by promoting ferroptosis and pyroptosis. Sci Rep. (2023) 13:14359. doi: 10.1038/s41598-023-41490-x, PMID: 37658132 PMC10474100

[98] Arnold M Abnet CC Neale RE Vignat J Giovannucci EL McGlynn KA . Global Burden of 5 Major Types of Gastrointestinal Cancer. Gastroenterology. (2020) 159:335–349.e315. doi: 10.1053/j.gastro.2020.02.068, PMID: 32247694 PMC8630546

[99] Kim R Taylor D Vonderheide RH Gabrilovich DI . Ferroptosis of immune cells in the tumor microenvironment. Trends Pharmacol Sci. (2023) 44:542–52. doi: 10.1016/j.tips.2023.06.005, PMID: 37380530

[100] Yang Z Su W Wei X Qu S Zhao D Zhou J . HIF-1α drives resistance to ferroptosis in solid tumors by promoting lactate production and activating SLC1A1. Cell Rep. (2023) 42:112945. doi: 10.1016/j.celrep.2023.112945, PMID: 37542723

[101] Zhang Y Yu R Li Q Song S Fu Y Shen X . Epigenetic suppression of Nrf2-Slc40a1 axis induces ferroptosis and enhances immunotherapy in pancreatic cancer. J Immunother Cancer. (2025) 13:e013269. doi: 10.1136/jitc-2025-013269, PMID: 41135950 PMC12557781

